# Phylogenetic Tools for Generalized HIV-1 Epidemics: Findings from the PANGEA-HIV Methods Comparison

**DOI:** 10.1093/molbev/msw217

**Published:** 2016-10-07

**Authors:** Oliver Ratmann, Emma B. Hodcroft, Michael Pickles, Anne Cori, Matthew Hall, Samantha Lycett, Caroline Colijn, Bethany Dearlove, Xavier Didelot, Simon Frost, A.S. Md Mukarram Hossain, Jeffrey B. Joy, Michelle Kendall, Denise Kühnert, Gabriel E. Leventhal, Richard Liang, Giacomo Plazzotta, Art F.Y. Poon, David A. Rasmussen, Tanja Stadler, Erik Volz, Caroline Weis, Andrew J. Leigh Brown, Christophe Fraser, on behalf of the PANGEA-HIV Consortium

**Affiliations:** 1 Department of Infectious Disease Epidemiology, MRC Centre for Outbreak Analyses and Modelling, School of Public Health, Imperial College London, London, United Kingdom; 2 School of Biological Sciences, Institute of Evolutionary Biology, University of Edinburgh, Edinburgh, United Kingdom; 3 The Roslin Institute, University of Edinburgh, Edinburgh, United Kingdom; 4 Nuffield Department of Medicine, Li Ka Shing Centre for Health Information and Discovery, Oxford Big Data Institute, University of Oxford, Oxford, United Kingdom; 5 Department of Mathematics, Imperial College London, London, United Kingdom; 6 Department of Veterinary Medicine, Cambridge Veterinary School, Cambridge, United Kingdom; 7 Department of Medicine, University of British Columbia, Vancouver, BC, Canada; 8 British Columbia Centre for Excellence in HIV/AIDS, Vancouver, BC, Canada; 9 Department of Environmental Systems Science, ETH Zürich, Zürich, Switzerland; 10 Department of Biosystems Science and Engineering, ETH Zürich, Basel, Switzerland; 11 Department of Civil and Environmental Engineering, Massachusetts Institute of Technology (MIT), Cambridge, MA; 12 Department of Pathology & Laboratory Medicine, Western University, Ontario, Canada

**Keywords:** HIV transmission and prevention, molecular epidemiology of infectious diseases, viral phylogenetic methods validation

## Abstract

Viral phylogenetic methods contribute to understanding how HIV spreads in populations, and thereby help guide the design of prevention interventions. So far, most analyses have been applied to well-sampled concentrated HIV-1 epidemics in wealthy countries. To direct the use of phylogenetic tools to where the impact of HIV-1 is greatest, the Phylogenetics And Networks for Generalized HIV Epidemics in Africa (PANGEA-HIV) consortium generates full-genome viral sequences from across sub-Saharan Africa. Analyzing these data presents new challenges, since epidemics are principally driven by heterosexual transmission and a smaller fraction of cases is sampled. Here, we show that viral phylogenetic tools can be adapted and used to estimate epidemiological quantities of central importance to HIV-1 prevention in sub-Saharan Africa. We used a community-wide methods comparison exercise on simulated data, where participants were blinded to the true dynamics they were inferring. Two distinct simulations captured generalized HIV-1 epidemics, before and after a large community-level intervention that reduced infection levels. Five research groups participated. Structured coalescent modeling approaches were most successful: phylogenetic estimates of HIV-1 incidence, incidence reductions, and the proportion of transmissions from individuals in their first 3 months of infection correlated with the true values (Pearson correlation > 90%), with small bias. However, on some simulations, true values were markedly outside reported confidence or credibility intervals. The blinded comparison revealed current limits and strengths in using HIV phylogenetics in challenging settings, provided benchmarks for future methods’ development, and supports using the latest generation of phylogenetic tools to advance HIV surveillance and prevention.

## Introduction

Recent breakthroughs in human immunodeficiency virus type 1 (HIV-1) prevention and treatment have provided a range of tools to reduce HIV-1 transmission (WHO 2015). Incorporating these strategies into routine care services and delivering on the commitment to end the HIV-1 epidemic by 2030 remains a major challenge (UNAIDS 2014), particularly in sub-Saharan Africa where the burden of HIV-1 is greatest. This region suffers 75% of all new HIV-1 infections worldwide, with adult HIV-1 prevalence exceeding 25% in some regions, and averaging ∼5% overall (UNAIDS 2015). To sustain public health interventions at this scale with limited resources, a sufficiently detailed understanding of the local and regional drivers of HIV-1 spread is often indispensable. Universal prevention packages (Iwuji et al. 2013; Hayes et al. 2014) benefit from data that allows monitoring incidence trends and drivers of residual spread, whereas more targeted prevention approaches (Vassall et al. 2014) by definition require a detailed knowledge of at-risk populations.

The Phylogenetics And Networks for Generalized HIV Epidemics in Africa (PANGEA-HIV) consortium aims to provide viral sequence data from across sub-Saharan Africa, and to evaluate their viral phylogenetic relationship as a marker of recent HIV-1 transmission dynamics (Pillay et al. 2015). Previous molecular epidemiological studies indicate that this approach can characterize transmission landscapes across a diverse array of epidemic contexts in order to guide prevention efforts (Fisher et al. 2010; Kouyos et al. 2010; von Wyl et al. 2011; Stadler et al. 2013; Volz et al. 2013; Grabowski et al. 2014; Bezemer et al. 2015; Ratmann et al. 2016). Rather than the partial gene sequences frequently used, the consortium is generating near full-length HIV-1 sequences in order to further increase the resolution and power of viral phylogenomic methods (Dennis et al. 2014). Indeed, such increases in power are needed to disentangle signal from noise in epidemic settings with frequent co-infection and recombination events (Grabowski et al. 2014), and to shift focus to recent transmission dynamics (Dennis et al. 2014).

Available viral phylogenetic techniques can provide estimates of key epidemiological quantities of concentrated HIV-1 epidemics (Brenner et al. 2007; Fisher et al. 2010; Stadler and Bonhoeffer 2013; Volz et al. 2013; Bezemer et al. 2015; Ratmann et al. 2016). But the generalized epidemics in sub-Saharan Africa and sequence availability in these resource-poor settings differ fundamentally from well sampled concentrated epidemics in wealthy countries, where viral phylogenetic tools have been proven to be most effective to date (Dennis et al. 2014). To strengthen the application of viral phylogenetics in sub-Saharan Africa, in October 2014 PANGEA-HIV invited research groups to participate in a blinded methods comparison exercise. Two individual-level HIV epidemic models were used to simulate generalized HIV-1 epidemics. From these, we generated corresponding viral sequence datasets comprising simulated *pol*, *gag* and *env* genes (which we refer to as full genome sequences for brevity), as well as basic individual-level epidemiological data on those infected individuals that were sequenced in the simulations. External research groups then analyzed the blinded data.

Overall, we aimed to evaluate if the most recent generation of viral phylogenetic tools could be adapted and used to estimate epidemiological quantities of central importance to HIV-1 prevention in sub-Saharan Africa. The specific objectives were inspired by current HIV-1 prevention trials in sub-Saharan Africa (Iwuji et al. 2013; Moore et al. 2013; Hayes et al. 2014). The primary goal of these trials is to achieve substantial reductions in HIV-1 incidence over a short period. Viral phylogenetics could be an effective tool to measure similar reductions, especially in contexts where incidence cohorts do not exist, and thereby contribute to monitoring the impact of prevention strategies. First, participants were asked to estimate recent reductions in HIV-1 incidence resulting from a simulated community-based intervention over a 3- to 5-year period. Here, incidence was defined as the proportion of new cases per year among uninfected adults, and reductions in incidence were measured in terms of the incidence ratio before and after the intervention. Second, it has been debated whether frequent transmission during the early acute phase of HIV infection could undermine the impact in reducing incidence of universal test and treat (Cohen et al. 2012). In concentrated epidemics, viral phylogenetics based on partial *pol* sequences have been used to provide estimates of the proportion of transmissions arising from individuals in their first year of infection (Volz et al. 2013; Ratmann et al. 2016). Here, we sought to evaluate whether viral phylogenetics based on full-genome sequences can provide accurate estimates of the proportion of transmissions from individuals in early and acute HIV (defined here as in their first 3 months of infection), because these are likely not preventable in current prevention trials where testing intervals are 1 year or more (Iwuji et al. 2013; Moore et al. 2013; Hayes et al. 2014). Third, as sequence data are now collected as part of HIV-1 prevention trials (HPTN 071 (PopART) Phylogenetics Protocol Team 2015; Novitsky et al. 2015), different approaches to prospective sequence sampling have emerged. Sequences could be collected at high coverage in villages or smaller townships at the risk of missing long-range transmissions, or at lower coverage over geographically much larger areas. We sought to compare the impact of these sampling strategies on viral phylogenetic analyses by simulating epidemics in village and larger regional populations, and sampling sequences at high and low coverage respectively. Other objectives included evaluating the benefit of using concatenated HIV-1 sequences comprising simulated *pol*, *gag* and *env* genes, as compared with using simulated *pol* sequences alone, and the impact of frequent viral introductions into the modeled population as a result of long-distance transmission. Table 1 describes the objectives and reporting variables of the exercise more fully.

**Table 1:** Aims of the PANGEA Phylodynamic Methods Comparison Exercise.

Objectives		Reporting Variable
**Primary objectives**		
1	Identify incident trends during the intervention	Consider the year $t_{s}\;$before the intervention started, and the second last year $t_{e}$ of the simulation. Participants were asked to report HIV-1 incidence trends from $t_{s}$ to $t_{e}$ in terms of “declining”, “stable”, “increasing”
2	Estimate HIV-1 incidence after the intervention	Participants were asked to report %Incidence defined as $\%INC(t_{e})=INC(t_{e})/S(t_{e})$, where $INC(t_{e})$ is the number of new cases in year $t_{e}$, and $S(t_{e})$ is the number of sexually active individuals that were not infected in year $t_{e}$
3	Quantify the reduction in HIV-1 incidence at the end of the intervention	Participants were asked to report the incidence ratio $\%INC(t_{e})/\%INC(t_{s})\;$
4	Estimate the proportion of transmissions from early and acute HIV, just before the intervention	Participants were asked to report the proportion of new cases in year $t_{s}$ from individuals in their first 3 months of infection
5	Estimate the proportion of transmissions from early and acute HIV, after the intervention.	Participants were asked to report the proportion of new cases in year $t_{e}$ from individuals in their first 3 months of infection
**Secondary objectives**		
To estimate the impact of the following controlled covariates on the reporting variables:		
6	Availability of full genome sequences (HIV-1 *gag*, *pol* and *env* genes) as compared with partial sequences (HIV-1 *pol* gene only)
7	Sequence sampling frame: Sequence coverage at the end of the simulation; Rapid increases in sequence coverage; Sampling duration after intervention start
8	Frequency of viral introductions into the modeled study population
9	Inference of dated viral phylogenies from sequence data

Five external research groups participated in the exercise, out of eight teams that initially indicated interest. Table 2 lists the phylogenetic methods that were used: the ABC-kernel method (A. Poon, J. Joy, R. Liang; team Vancouver) (Poon 2015), the birth-death skyline method with sampled ancestors (C. Weis, G.E. Leventhal, D. Kühnert, D.A. Rasmussen, T. Stadler; team Basel-Zürich) (Gavryushkina et al. 2014; Kühnert et al. 2016), a metapopulation coalescent approach (B. Dearlove, M. Hossain, S. Frost; team Cambridge) (Dearlove and Wilson 2013), the structured coalescent (E. Volz, M. Hossain, S. Frost; team Cambridge-London) (Volz et al. 2009), and a Bayesian transmission chain analyser (C. Colijn, M. Kendall, X. Didelot, G. Plazotta; team London) (Didelot et al. 2014). These methods differed in the underlying transmission and intervention models, assumptions to facilitate estimation of the reporting variables, and computational estimation routines. Here, we summarize the findings of the exercise, and discuss their implications for using phylogenetic methods to estimate recent aspects of HIV-1 transmission dynamics in generalized epidemics. Datasets and simulations generated here may be of use for testing other applications of viral phylogenetic methods, and are made available alongside this article.

**Table 2:** Phylogenetic Methods Used in the PANGEA Phylodynamic Methods Comparison Exercise.

Team	Team Members	Method	Model-based analysis	Model Overview	Simulated Data Used To Inform Inference	Fitting Process	Availability
Basel-Zürich	C. Weis, G.E. Leventhal, D. Kühnert, D.A. Rasmussen, T. Stadler	Birth–death skyline method with sampled ancestors	Yes	Stochastic birth–death model with sampled ancestors to estimate incidence and incidence reductions, and multi-type birth death model corresponding to two stages of infection to estimate the proportion of early transmissions. Time trends in parameters were modeled with serial time intervals during which parameters were assumed constant. Viral introductions were not modeled	All sequences and full trees to estimate birth–death parameters; cross-sectional survey data	Markov Chain Monte Carlo	http://beast2.org/ (last accessed October 14, 2016) using add-ons bdsky, SA, bdmm
Cambridge	B. Dearlove, M. Hossain, S. Frost	Meta-population coalescent approach	Yes	Standard SI, SIS and SIR models were averaged. Model parameters did not change over time. Viral introductions were not modeled.	All sequences and full trees.	Markov Chain Monte Carlo	http://beast.bio.ed.ac.uk/ (last accessed October 14, 2016) using XML specification described in (Dearlove and Wilson 2013)
Cambridge-London	E. Volz, M. Hossain, S. Frost	Structured coalescent	Yes	Deterministic compartment model stratified by gender, disease progression, diagnosis and treatment status, risk behavior. Time trends in baseline transmission rates were modeled with 4-parameter generalized logistic function. Diagnosis and treatment uptake rates changed at intervention start. Viral introductions were modeled with a source deme.	All sequences and sub-trees including all internal nodes 30 before the last sample; cross-sectional survey data; and gender and CD4 count at time of diagnosis for Regional datasets.	Parallel Markov Chain Monte Carlo	http://colgem.r-forge.r-project.org/ (last accessed October 14, 2016)
London	C. Colijn, M. Kendall, G. Plazotta, X. Didelot	Bayesian transmission chain analyzer	Yes	Stochastic generalized branching model with generation time modeled to represent three infection stages. Model parameters did not change over time. Viral introductions were not modeled.	All sequences and full trees on village datasets; sequences in trees with at least 80 tips on regional datasets.	Reversible-jump Markov Chain Monte Carlo	https://github.com/xavierdidelot/TransPhylo (last accessed October 14, 2016) PANGEA release available from authors
Vancouver	A. Poon, J. Joy, R. Liang	ABC kernel method	Yes	Deterministic compartment model stratified by infection status, three stages of infection, and risk behavior. Model parameters did not change over time. Viral introductions were modeled with a source deme.	All sequences and full trees.	Approximate Bayesian Computation	https://github.com/ArtPoon/kamphir (last accessed October 14, 2016) PANGEA release available from authors

## Results

### PANGEA-HIV Reference Datasets for Benchmarking Molecular Epidemiological Transmission Analysis Methods

The simulations capture a variety of transmission and intervention scenarios across two demographic settings in sub-Saharan Africa, and are available from https://dx.doi.org/10.6084/m9.figshare.3103015 (last accessed October 14, 2016).

**Fig. 1:**
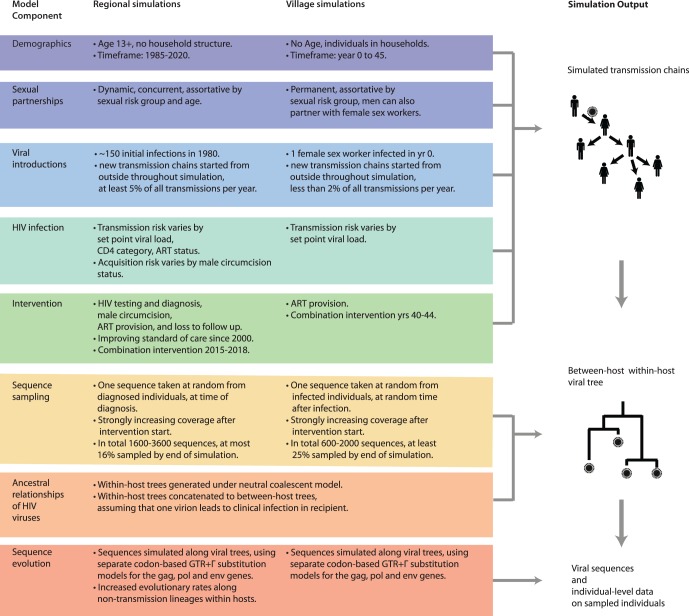
Simulation pipeline to generate HIV-1 sequence data, viral phylogenies, and accompanying individual-level data. Two simulation models (Regional and Village) were implemented for the methods comparison. The two individual-level epidemic and intervention models generated HIV-1 transmission chains in the model population, and its components are shown in blue to green. Next, individuals were sampled for sequencing, and a viral tree was generated for these individuals. Tree generation accounted for within-host viral evolution under a neutral coalescent model. Finally, viral sequences comprising the *gag*, *pol* and *env* genes were simulated along the viral tree. Sequence generation accounted for known variation in evolutionary rates across genes, codon positions, and along within-host lineages. Further details are provided in supplementary tables S1 and S2, Supplementary Material online.

**Fig. 2:**
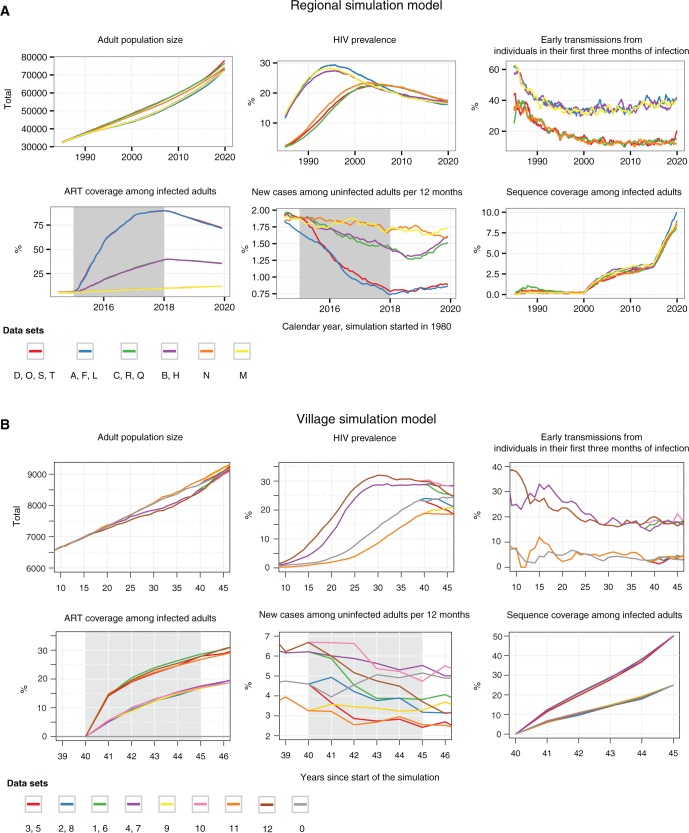
Simulated epidemic scenarios under the Regional and Village models. (*A*) Six generalized HIV-1 epidemic scenarios were simulated in a region of ∼80,000 adult individuals using the Regional model, and (*B*) nine scenarios were simulated in a rural village population with an initial population of ∼6,000 individuals using the Village model. The scenarios differ in terms of incidence, the proportion of early transmissions, and scale-up of the combination prevention package during the intervention period (gray-shaded time period). From these, 33 datasets were generated, that included either viral sequences or viral trees. These datasets further varied in the sequence sampling frame and the frequency of viral introductions; see also figure 1 and table 3. Datasets E, G, I, J, K, P had more frequent viral introductions or higher sequence coverage, and are not shown. The proportion of early transmissions under the Village model was smoothed with a 3-year sliding window to better visualize trends in this smaller model population.

20 datasets correspond to generalized HIV-1 epidemics in a region of ∼80,000 individuals between 1980 and 2020 (table 3). The proportion of infected individuals of whom one sequence was sampled (sequence coverage) was 8–16% by the end of the simulation. These data were simulated under the individual-based HPTN071 (PopART) model, version 1.1, developed at Imperial College London (“Regional” model). The overall simulation pipeline and model components are illustrated in figure 1, and further information is provided in supplementary table S1, Supplementary Material online. The Regional model was calibrated to generate an epidemic with a comparable prevalence at the start of the intervention to that seen currently in HPTN071 (PopART) trial sites in South Africa (Hayes et al. 2014). In the model, standard of care improved according to national guidelines over time, resulting in steady declines in incidence. In 18 of the 20 simulations, a combination prevention intervention was started in 2015 for 3 years at varying degrees of uptake and coverage, resulting in 30% or 60% reductions in incidence relative to the start of the intervention, when incidence was close to 2% per year. In half of the 20 simulations, the proportion of early transmissions in 2015 was respectively calibrated to 10% and 40% (fig. 2). Ranges in incidence reduction reflect modeled, optimistic and pessimistic scenarios in on-going prevention trials in sub-Saharan Africa (Iwuji et al. 2013; Moore et al. 2013; Hayes et al. 2014). The proportion of transmissions from early and acute HIV has been challenging to estimate without sequence data, and the ranges used here reflect estimates from several settings in sub-Saharan Africa (Cohen et al. 2012). About 5–20% of all transmissions per year occurred from outside the model population, which hindered prevention efforts in the simulations through continual replenishment of the epidemic.

**Table 3:** Simulated Datasets of the Phylodynamic Methods Comparison Exercise.

Model	Dataset	Purpose	%Acute^a^^,^^b^ (Low=L, High=H)	Intervention Scale Up^a^^,^^c^ (Fast=F, Slow=S)	Viral Introductions^a^^,^^d^ (% of All Transmissions per Year)	Sequences (#)	Sequence Coverage in the Last Year of the Simulation^a^^,^^e^ (% of All Infected and Alive)	Sequences After Intervention Start^f^ (% of All Sequences)	Sampling Duration After Intervention Start^g^ (Years)
**Regional**^h^									
	**D**	Identify 60% reduction in incidence during intervention and 10% early transmissions.	L	F	5	1,600	8	50	5
	**C**	Identify 30% reduction in incidence during intervention and 10% early transmissions.	L	S	5	1,600	8	50	5
	**A**	Identify 60% reduction in incidence during intervention and 40% early transmissions.	H	F	5	1,600	8	50	5
	**B**	Identify 30% reduction in incidence during intervention and 40% early transmissions.	H	S	5	1,600	8	50	5
	O	As D, and evaluate impact of sampling frame: shorter duration of intensive sequencing.	L	F	5	1,280	8	50	3
	T	As D, and evaluate impact of tree reconstruction.	L	F	5	1,600	8	50	5
	S	As D, and evaluate impact of sampling frame: most sequences from after intervention start.	L	F	5	1,600	8	85	5
	I	As D, and evaluate impact of sampling frame: higher sequence coverage.	L	F	5	3,200	16	50	5
	R	As C, and evaluate impact of tree reconstruction.	L	S	5	1,600	8	50	5
	Q	As C, and evaluate impact of sampling frame: most sequences from after intervention start.	L	S	5	1,600	8	85	5
	G	As C, and evaluate impact of sampling frame: higher sequence coverage.	L	S	5	3,200	16	50	5
	N	Control simulation, no intervention.	L	None	5	1,600	8	50	5
	F	As A, and evaluate impact of sampling frame: shorter duration of intensive sequencing.	H	F	5	1,280	8	50	3
	L	As A, and evaluate impact of tree reconstruction.	H	F	5	1,600	8	50	5
	J	As A, and evaluate impact of sampling frame: higher sequence coverage.	H	F	5	3,200	16	50	5
	P	As A, and evaluate impact of higher proportion of viral introductions.	H	F	20	1,600	8	50	5
	H	As B, and evaluate impact of tree reconstruction.	H	S	5	1,600	8	50	5
	K	As B, and evaluate impact of sampling frame: higher sequence coverage.	H	S	5	3,200	16	50	5
	E	As B, and evaluate impact of higher proportion of viral introductions.	H	S	20	1,600	8	50	5
	M	Control simulation, no intervention.	H	None	5	1,600	8	50	5
**Village**^h^									
	**3**	Identify 40% reduction in incidence during intervention and 4% early transmissions.	L	F	<2	777	25	>95	5
	**2**	Identify 15% reduction in incidence during intervention and 4% early transmissions.	L	S	<2	857	25	>95	5
	**1**	Identify 40% reduction in incidence during intervention and 20% early transmissions.	H	F	<2	957	25	>95	5
	**4**	Identify 15% reduction in incidence during intervention and 20% early transmissions.	H	S	<2	1,040	25	>95	5
	5	As 3, and evaluate impact of sampling frame: higher sequence coverage.	L	F	<2	1,469	50	>95	5
	11	Similar to 3, without imported sequences.	L	F	0	638	25	>95	5
	8	As 2, and evaluate impact of sampling frame: higher sequence coverage.	L	S	<2	1,630	50	>95	5
	9	Similar to 2, without imported sequences.	L	S	0	686	25	>95	5
	0	Control simulation, no intervention.	L	None	<2	872	25	>95	5
	6	As 1, and evaluate impact of sampling frame: higher sequence coverage.	H	F	<2	1,831	50	>95	5
	12	Similar to 1, without imported sequences.	H	F	0	956	25	>95	5
	7	As 4, and evaluate impact of sampling frame: higher sequence coverage.	H	S	<2	1,996	50	>95	5
	10	Similar to 4, without imported sequences.	H	S	0	1,012	25	>95	5

a Variables in shaded columns were unknown to participants at time of analysis.

b Values range from 5% to 40%, reflecting recent estimates for endemic-phase epidemics in sub-Saharan Africa (Cohen et al. 2012).

c Range reflects optimistic and pessimistic scenarios in prevention trials in sub-Saharan Africa (Iwuji et al. 2013; Moore et al. 2013; Hayes et al. 2014).

d Range includes frequent viral introductions as reported in settings with highly mobile populations (Grabowski et al. 2014).

e In comparison to the large sequence datasets that are available for concentrated epidemics in Europe or North America, the lower values here reflect challenges in achieving high sequence coverage where large populations are infected. Higher values reflect geographically focused sequencing efforts such as in Mochudi, Botswana (Carnegie et al. 2014).

f Values reflect the duration of typical prevention trial settings, and that most sequences are obtained after intervention start (Iwuji et al. 2013; Moore et al. 2013; Hayes et al. 2014).

g Out of all individuals that were alive and infected in the last calendar year of the simulation, the proportion that had ever a sequence taken.

h For datasets in bold, only viral sequences were disclosed. For all other datasets, only viral phylogenies were provided.

13 simulated datasets capture generalized HIV-1 epidemics over 45 years in a smaller village population of ∼8,000 individuals (table 3). Sequence coverage was higher in this smaller population, 25–50% by the end of the simulation. These data were simulated under an individual-based household model using the Discrete Spatial Phylo Simulator for HIV, developed at the University of Edinburgh (“Village” model). Model components are illustrated in figure 1, and further information is provided in supplementary table S2, Supplementary Material online. The Village model was parameterized to simulate an HIV-1 epidemic mostly contained within a small rural African village, with a peak prevalence of 20–25% and peak incidence of 5–7% without treatment (fig. 2). In 12 out of 13 simulations, a community-level intervention providing antiretroviral treatment took place for the last 5 years of the simulation. Treatment uptake was either “fast” or “slow”, with reductions in incidence averaging between 10% and 40% relative to before intervention start. Additionally, simulations were configured so that either a small (4%) or large (20%) proportion of transmissions occurred during the first 3 months of infection. Some infections originated from outside the model population in half of the simulations.

Viral sequences were generated from the simulated transmission chains (fig. 1). First, individuals were sampled at random for sequencing. The majority of individuals were only sampled in the last years of the simulations, reflecting that sequences are only beginning to be more routinely collected in sub-Saharan Africa (Iwuji et al. 2013; Moore et al. 2013; Dennis et al. 2014; Grabowski et al. 2014; HPTN 071 (PopART) Phylogenetics Protocol Team 2015; Pillay et al. 2015). Sequence sampling biases can be substantial in real datasets, but were not included in the model (Carnegie et al. 2014; Ratmann et al. 2016). Second, viral trees were generated under a hybrid within- and between-host coalescent model. The viral trees did not always correspond to the transmission trees, because viruses diversified within infected individuals before transmission (Pybus and Rambaut 2009). In 25 of the 33 datasets, these viral trees were made available, in order to reduce the computational burden of molecular epidemiological analyses (table 3 and supplementary figs. S1 and S2, Supplementary Material online). For the remaining 13 datasets, viral sequences of HIV-1 *gag*, *pol* and *env* genes were simulated along the viral trees (∼1,500, ∼3,000 and ∼2,500 nucleotides respectively, for a total of approximately 6,000 nucleotides), from an HIV-1 subtype C starting sequence. The sequences thus represent generalized subtype C epidemics, as in most Southern African countries. The nucleotide sequence evolution model that was used incorporated known differences in evolutionary rates by gene and codon position and relative differences in substitution rates by gene and codon position (Shapiro et al. 2006; Alizon and Fraser 2013). The coalescent and sequence evolution models did not account for recombination, sequencing errors, or selection beyond differential evolutionary rates across genes, codons and within-host lineages (supplementary tables S1 and S2, Supplementary Material online). As a key indicator of the realism of the simulated sequences, we calculated the proportion of the variation in evolutionary diversification among the simulated HIV-1 sequences, that can be explained by a constant molecular clock model. The proportion explained ranged from 25% to 60% (supplementary figs. S3 and S4, Supplementary Material online), broadly in line with estimates on real HIV-1 sequence datasets (Lemey et al. 2006).

The simulations were designed to retain signal for differentiating between the “fast”, “slow” and “no” community-level intervention scenarios through the viral sequences provided (supplementary fig. S5, Supplementary Material online). However, we expected that rapid increases in sequence coverage after the intervention would complicate phylogenetic inference. The simulations also retained, on average, information for differentiating between the 10% and 40% early transmission scenarios of the Regional simulations at very low sequence coverage (supplementary fig. S6, Supplementary Material online). More challenges were expected on the Village simulations despite higher sequence coverage, partly because the effect size between the low and high %Acute scenarios was smaller (supplementary fig. S7, Supplementary Material online).

### Responses to the Methods Comparison Exercise

Participants were primarily asked to estimate incidence reductions from before the intervention (year 39 or 2014) to just after the intervention (year 43 or 2018), and to estimate the proportion of early transmissions in the year before and after the intervention (table 1). Participating teams developed fast computational strategies for handling full-genome HIV sequence datasets within given timelines (3 months for 13 Village datasets and 6 months for 20 regional datasets). First, where only sequences were provided, viral phylogenies were reconstructed with maximum likelihood methods (Price et al. 2010; Stamatakis 2014). Second, these phylogenies were dated under least-squares criteria or similar fast approaches (To et al. 2015). Third, dated phylogenies were used as input to the transmission analysis methods described in table 2. This sequential approach allowed the teams to obtain phylogenetic estimates to all reporting variables for the large majority of the datasets (see supplementary table S3, Supplementary Material online). Team Vancouver did not provide estimates to datasets of the Regional model that contained true phylogenetic trees; and teams Cambridge-London and Basel-Zürich did not provide estimates to datasets of the Regional model that contained sequences. The most common reasons for incomplete recall were limited availability of computing resources, tight timelines to evaluate the simulations, and difficulties in tree estimation when viral introductions occurred frequently. Nearly all participants focused on inference from full viral genomes (supplementary table S3, Supplementary Material online), meaning that the impact of full genome sequences (concatenated HIV-1 *gag*, *pol* and *env* genes) as compared with partial sequences (HIV-1 *pol* gene only) could not be evaluated.

### Estimating Incidence and Reductions in Incidence

**Fig. 3:**
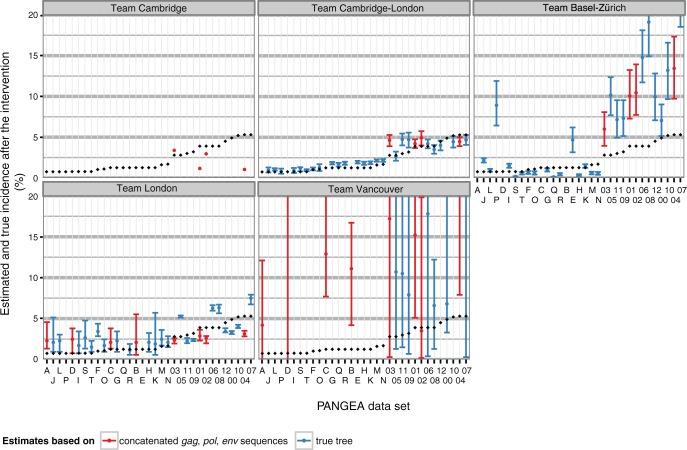
Estimates of HIV-1 incidence from phylogenetic methods on simulated PANGEA datasets. Submitted estimates are shown for each PANGEA dataset by research team (panel) and type of data provided (either sequences or the viral phylogenetic tree, color). Error bars correspond to 95% credibility or confidence intervals. True values are shown in black.

Phylogenetic methods differed in their ability to estimate incidence after the intervention (fig. 3). Under the most successful computational approach, phylogenetic estimates of incidence were correlated with true values by 91% (supplementary table S2, Supplementary Material online, team Cambridge-London who used a structured coalescent model). Bias in these estimates was relatively small for estimates of two teams (on an average 0.35% by team Cambridge-London and 0.57% by team London). Team Basel-Zürich achieved substantially more accurate estimates on the Regional datasets than the Village datasets, whereas the converse was true for team London (supplementary table S2, Supplementary Material online).

**Fig. 4:**
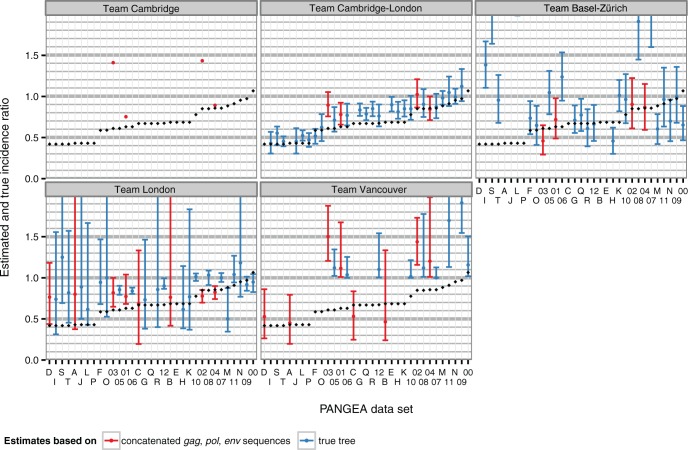
Estimates of HIV-1 incidence reductions from phylogenetic methods on simulated PANGEA datasets. Submitted estimates are shown for each PANGEA dataset by research team (panel) and type of data provided (either sequences or the viral phylogenetic tree, color). Error bars correspond to 95% credibility or confidence intervals. True values are shown in black.

The accuracy of phylogenetic estimates of changes in incidence as a result of the intervention largely reflected the accuracy of the underlying incidence estimates (fig. 4). Phylogenetic estimates of incidence ratios correlated with the true values by 93% under the structured coalescent approach of team Cambridge-London, and had only slight upward bias (supplementary table S4, Supplementary Material online). This meant that large reductions in incidence, which are expected from combination prevention interventions, could be correctly detected at relatively low sequence coverage when sequences were sampled for 5 years since intervention start by the most successful method. Epidemic simulations with >25% reductions in incidence were correctly classified as declining in 15/17 (88%) of all simulations with a submission by team Cambridge-London, although the true positive rate was lower with other phylogenetic methods (supplementary table S5, Supplementary Material online).

### Estimating the Proportion of Transmissions from Individuals in Their First Three Months of Infection (Early and Acute HIV)

**Fig. 5:**
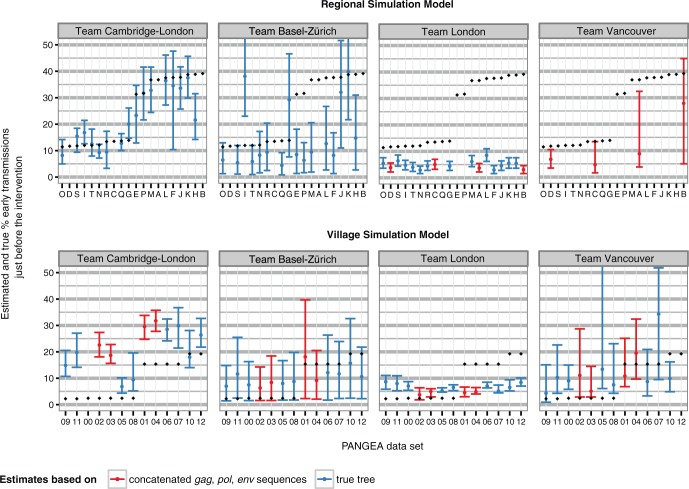
Estimates of the proportion of transmissions from individuals in their first 3 months of infection (early and acute HIV), before the intervention from phylogenetic methods on simulated PANGEA datasets. Submitted estimates are shown for each PANGEA dataset by research team and model simulation (panels) and type of data provided (either sequences or the viral phylogenetic tree, color). Error bars correspond to 95% credibility or confidence intervals. True values are shown in black.

Phylogenetic estimates of the proportion of early transmissions just before and after the intervention were more accurate on the Regional simulations than the Village simulations, potentially reflecting stronger signal as a result of larger effect sizes in the Regional simulations (fig. 5 and supplementary figs. S6–S8, Supplementary Material online). On the regional simulations, estimates by team Cambridge-London had a mean absolute error of 3.9% and correlated with true values by 92%. However, on the Village simulations, the mean absolute error in estimates by team Cambridge-London was 12% (supplementary table S6, Supplementary Material online). Other teams had, overall, difficulties recovering the frequent early transmission scenarios. Team Basel-Zürich achieved the smallest mean absolute error on the Village simulations (supplementary table S6, Supplementary Material online).

### Predictors of Large Error in Phylogenetic Estimates

**Fig. 6:**
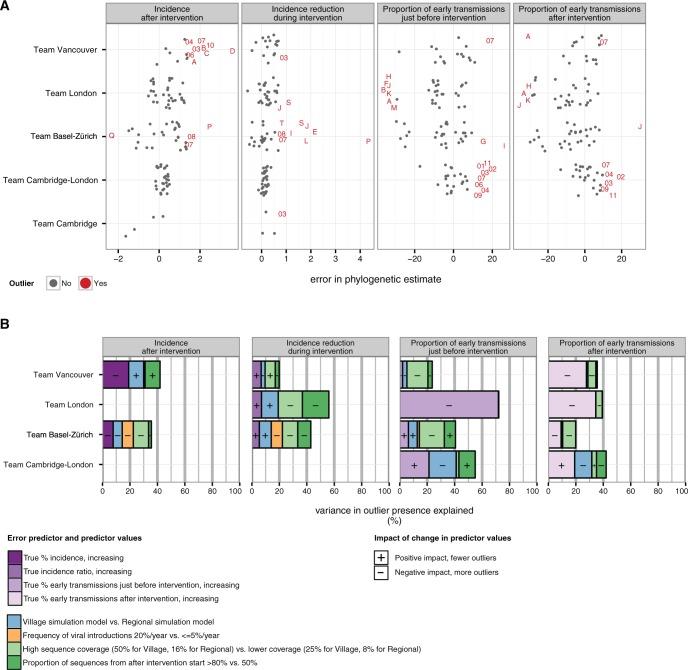
Predictors of large error in phylogenetic estimates. (*A*) For each response, the error in the phylogenetic estimate was calculated, and statistical outliers were identified. The plot shows error in phylogenetic estimates by team and outcome measure. For large errors, the corresponding PANGEA dataset code in table 1 is indicated. (*B*) The contribution of the systematically varied covariates in table 1 to the presence of outliers was quantified through partial least squares regression (PLS, see “Materials and Methods” section). The plot shows the contribution of each predictor to the variance in outlier presence in colors, and the corresponding signs of the regression coefficients are added. Estimates from team Cambridge could not be characterized due to small sample size. The impact of the error predictors varied across the primary objectives of phylogenetic inference, as well as the phylogenetic methods used. With regard to estimates of incidence and incidence reduction, a subset of phylogenetic methods was particularly sensitive to high sequence coverage, a very large proportion of sequences obtained after intervention start, and a large frequency of viral introductions. With regard to estimates of the proportion of early transmissions, outliers were in several cases best explained by true differences in the proportion of early transmissions.

We evaluated to what extent the variation in errors of phylogenetic estimates could be associated to systematic differences in the simulation datasets (referred to as “covariates”), such as sequence coverage and frequency of viral introductions (table 3). Figure 6A illustrates the phylogenetic estimates that deviated largely from the true values (referred to as “outliers”). We focused on quantifying the association of outlier presence with the covariates listed in table 3 using a partial least squares regression approach, which enabled us to handle a relatively large number of co-dependent covariates (see “Materials and Methods” section).

Several covariates could be excluded from this analysis. Estimates obtained from the simulated full genome sequence datasets were not more strongly associated with estimation error than estimates obtained using the phylogenetic trees from which the sequences were simulated (supplementary fig. S9 and supplementary table S7, Supplementary Material online). Shorter, intense sampling periods after intervention start of 3 years compared with a default of 5 years were also not strongly associated with larger estimation error (supplementary table S7, Supplementary Material online).

Figure 6B shows the proportion of variance in outlier presence that is explained by each of the remaining covariates. Signs indicate the impact of a change in predictor values on the number of phylogenetic estimates with very large error. Subplots are empty when phylogenetic methods did not produce estimates with large error (indicating a higher degree of success). Overall, with regard to estimates of incidence and incidence reduction, higher sequence coverage (16% vs. 8% in the Regional datasets and 50% vs. 25% in the Village datasets) and a large proportion of sequences obtained after intervention start (>80% vs. 50%) were associated with more outliers for more than one phylogenetic method. Frequent viral introductions (20%/year vs.  < =5%/year) were associated with more outliers by team Basel-Zürich. These predictors tended to outweigh the impact that true differences in incidence and incidence reduction had on outlier presence.

In contrast, with regard to estimates of the proportion of early transmissions, outliers were in several cases best explained by true differences in the proportion of early transmissions. Several phylogenetic methods had substantial difficulty estimating frequent early transmissions. Low sampling coverage did not contribute substantially to the presence of outliers. To substantiate this observation further, we compared phylogenetic estimates from just before the intervention to those after the intervention, and found no consistent improvements in accuracy with a doubling of sampling coverage (supplementary fig. S10, Supplementary Material online). Instead, outlier presence could be explained through the simulation model, with more outliers on the Village datasets. These simulations were characterized by smaller sample sizes and smaller effect size (table 3 and supplementary figs. S6 and S7, Supplementary Material online).

## Discussion

The PANGEA methods comparison exercise represents a community-wide effort for advancing the use of phylogenetic methods to estimate aspects of recent HIV-1 transmission dynamics of generalized epidemics in sub-Saharan Africa. This region is affected by the largest HIV-1 epidemics worldwide. Viral phylogenetics could be a central tool to guide HIV-1 prevention in these settings (Dennis et al. 2014).

It is not possible for phylogenetic methods to capture all factors that influence the spread of HIV-1, ranging all the way from biological factors determining person-to-person transmission (Cohen et al. 2011) to the structure of sexual networks on the community level (Gregson et al. 2002; Tanser et al. 2011), and the broader impact of prevention and care services (Gardner et al. 2011). Of course, capturing all such features may not be needed: particular aspects of HIV-1 spread in generalized epidemics could be estimable from sequence data under the simplifying assumptions of phylogenetic methods, and at relatively low sequence coverage.

To validate this hypothesis from the outset, the PANGEA-HIV team simulated data under two highly complex HIV transmission and intervention models, whose components are considered essential for understanding long-term HIV transmission dynamics (Eaton et al. 2012). The aspects of HIV-1 spread evaluated here (table 1) were chosen both because molecular epidemiological studies into the sources of transmission and temporal changes in epidemic spread are in principle feasible (von Wyl et al. 2011; Stadler et al. 2013; Volz et al. 2013; Dennis et al. 2014; Ratmann et al. 2016), and because of their relevance to on-going HIV-1 prevention efforts in sub-Saharan Africa. Crucially, the model simulations were constrained to pessimistic and optimistic projections of the likely outcomes of on-going HIV-1 prevention efforts in sub-Saharan Africa (Iwuji et al. 2013; Moore et al. 2013; Hayes et al. 2014), as well as what sequence data could become available in these settings.

The methods comparison exercise was challenging. First, the exercise focused on quantifying recent transmission dynamics, whereas HIV-1 sequence data are more routinely used to characterize the origins and spread of the virus (Faria et al. 2014), or to undertake descriptive analyses of putative transmission chains (Brenner et al. 2007; Dennis et al. 2012). To be precise, the challenge here was in obtaining quantitative estimates of HIV-1 incidence and the sources of transmission in generalized epidemics, and to do so close to the present, when the phylogenetic signal weakens (de Silva et al. 2012). Second, sequence coverage was relatively low in most simulations, as is expected for most endemic-phase settings in sub-Saharan Africa. Furthermore, frequent viral introductions complicated the interpretation of viral trees, timelines were tight (3 months for the Village datasets, and 6 months for the Regional datasets), and phylodynamic models had to represent viral spread in heterogeneous populations (males and females with different risk profiles). We aspired to evaluate the extent to which these challenges can be addressed with full genome HIV-1 sequences, and through customized phylogenetic methods.

The methods comparison exercise demonstrates that viral phylogenetic tools can successfully estimate aspects of recent transmission dynamics of generalized HIV-1 epidemics at limited sequence coverage of the infected population, when full-genome sequences are available. Two methods, the ABC kernel method of team Vancouver and the Bayesian transmission analyzer of team London (table 2), were newly developed in response to the exercise. The birth–death skyline model with sampled ancestors (Gavryushkina et al. 2014) and its multi-type analogue (Kühnert et al. 2016) are readily available through the BEAST2 software package. The structured coalescent (Volz et al. 2009) was customized to reflect available information on the simulated epidemics, and required considerable resources (roughly 1 week of computation time on a 64-core machine of 2.5 Ghz processors per analysis). The methods comparison reflects these different stages in development and customization. In this context, the structured coalescent approach was overall most accurate, producing accurate estimates of incidence and changes in incidence, as well as broadly accurate estimates into the proportion of early transmissions on the Regional simulations from full-genome sequences. Confidence intervals were sufficiently tight for epidemiological interpretation, bearing in mind that uncertainty in tree reconstructions was ignored. This indicates that the latest generation of viral phylogenetic methods can complement standard incidence estimation techniques where full-genome sequences are available from the general population. The use of sequence data for estimating incidence trends in sub-Saharan Africa could be particularly useful where demographic and health survey data are sparse (Pillay et al. 2015), no relevant observational HIV cohorts exist, or where estimates would otherwise be solely reliant on data from particular population groups such as pregnant women (Montana et al. 2008). Further, this study supports using viral phylogenetic methods for identifying sources of HIV-1 transmission from full-genome sequences in certain settings. Broadly accurate estimates into the fraction of transmissions attributable to a population group were obtained when both transmission from that group was not infrequent (at least 10%) and sample size was not too small (thousands of sequences for the HIV-infected populations considered). Viral phylogenetic methods could thus help to quantify the contribution of several other source populations that are of key interest for prevention in sub-Saharan Africa, including the proportion of individuals infected within localized high prevalence areas (Tanser et al. 2013), or the proportion of young women infected by male peers (Dellar et al. 2015).

We varied aspects of transmission dynamics and the sampling frame in the simulations, to obtain a more systematic understanding of methods’ performance (fig. 5). Most phylogenetic methods did not identify significant differences between the high/low early transmission scenarios, and this was also the case when basic genetic distance measures recovered differences between the high/low early transmission scenarios (regional simulations, supplementary fig. S6, Supplementary Material online). The true proportions of early transmissions were also frequently outside 95% confidence or credibility intervals. This indicates that further methods’ improvement is needed for estimating the proportion of early transmissions, and potentially for attributing sources of HIV-1 transmission more broadly at the low sequence coverage scenarios considered. Further, nearly all participants reported difficulties in achieving numerical convergence of their methods on full-genome sequence data (unpublished submission reports). This could explain the above observations in part, and in particular why the accuracy of early transmission estimates did not improve when using larger datasets with higher sequence coverage (fig. 5 and supplementary fig. S10, Supplementary Material online). Further investigations are needed. Finally, our error analysis suggests that explicit modeling of unobserved source demes (team Cambridge-London) or identification of spatially localized phylogenetic clusters prior to transmission analyses (team London) could be effective approaches for mitigating the negative impact of viral introductions on phylogenetic analyses on mobile populations (Grabowski et al. 2014). The simulated PANGEA datasets as well as various aspects of the corresponding true epidemics and interventions are available for future benchmarking.

This study has limitations. First, phylogenetic methods were evaluated on simulated HIV-1 epidemics. While the use of two models guards to some extent against over-interpretation, analyses of real datasets may be more complex and could be associated with overall larger error. Of note, the simulated datasets are free of sequence sampling biases, which can substantially distort phylogenetic inferences (Carnegie et al. 2014). Second, the evolutionary components of the two models generated sequences that do not contain gaps or sequencing errors, cannot be translated to amino acids, were correctly aligned, and did not contain recombinant sequences. Viral trees reconstructed from real sequence data are likely less accurate than those used in this analysis, a potential source of error that is not represented in our evaluations. Frequent recombination could imply that full HIV-1 genomes are more appropriately analyzed on a gene-by-gene basis (Hollingsworth et al. 2010; Ward et al. 2013), in contrast to our full-genome analyses of simulated sequences that excluded recombinants. This limitation is particularly relevant to epidemic settings in sub-Saharan Africa where multiple subtypes and recombinant forms circulate at high frequencies. Third, phylogenetic analyses of full-genome sequences were not compared with similar analyses using shorter fragments of the genome such as, e.g., several 250 base pair regions from the *gag*, *pol* or *env* genes. Full-genome sequences may not be required for estimating recent changes in HIV-1 incidence or for quantifying the sources of HIV-1 transmission, and more cost-effective sequencing approaches could provide similar results.

The PANGEA-HIV methods comparison exercise showed viral phylogenetic methods can be adapted to provide quantitative estimates on aspects of recent HIV-1 transmission dynamics in sub-Saharan Africa, where sequence coverage remains limited. On simulations, the structured coalescent approach was overall most accurate for estimating recent changes in incidence and the proportion of early transmissions in modeled populations with generalized, and large HIV-1 epidemics. Future molecular epidemiological analyses would ideally make use of several of the evaluated phylogenetic tools, in order to obtain robust insights into HIV-1 transmission flows and how to disrupt them. Further methods’ refinement is required to this end, with our analysis suggesting a focus on estimating the sources of HIV-1 transmission from full-genome HIV-1 sequence data. These findings were obtained through a community-wide, blinded evaluation, and thereby add confidence into the use and interpretation of viral phylogenetic tools for HIV-1 surveillance and prevention in sub-Saharan Arica and beyond.

## Materials and Methods

### Study Design

The blinded PANGEA-HIV methods comparison exercise was announced in October 2014 at HIV Dynamics & Evolution, and later on the PANGEA-HIV website. In a training round (round 1), participants were asked to identify trends in incidence on simulated sequence datasets that were similar in size to the datasets in table 3, but that had qualitatively different epidemic dynamics. Data included full-genome viral sequences, patient meta-data, and further broad information on the simulated epidemic (supplementary text S1, Supplementary Material online). Participation was unrestricted. In December 2014, the training data were un-blinded. All participants shared their findings. PANGEA-HIV and the participants agreed on the objectives and reporting variables listed in table 1; on the timelines for the second final round; and that participation will be retrospectively restricted to teams addressing at least one of the pre-specified reporting variables. Simulation models were updated to include explicit HIV care and intervention components, and re-calibrated to generate the epidemic scenarios shown in figures 1 and 2. Blinded datasets were released on 10 February 2015 (supplementary text S2, Supplementary Material online). The deadline for submissions was 8 May 2015. Questions and clarifications during the exercise were disseminated to all participants. Submissions were checked manually, and teams were given the opportunity to fix conceptual errors. Few submissions to the Regional simulations were obtained, and the deadline for submission to Regional datasets was extended to 18 August 2015. The Village simulations were un-blinded on 14 May 2015, and a preliminary evaluation was presented and reviewed by all participants at the 22nd HIV Dynamics & Evolution conference. Teams Vancouver and Basel-Zürich informed the evaluation group of a conceptual misunderstanding of the reporting variables, and provided updated incidence estimates after the intervention 1 day after the presentation. These updates on the Village datasets were used in the evaluation reported here. The Regional datasets were un-blinded on 3 September 2015.

### Village Simulations

The Village simulations were generated using the Discrete Spatial Phylo Simulator with HIV-specific components (DSPS-HIV, https://github.com/PangeaHIV/DSPS-HIV_PANGEA; last accessed October 14, 2016). The DSPS-HIV is an individual-based stochastic simulator which models HIV-1 transmissions along a specifiable contact network of individuals and produces a line-list of all events (Hodcroft 2015). Viral phylogenies that reflect between- and within-host viral evolution were generated along transmission chains using VirusTreeSimulator (https://github.com/PangeaHIV/VirusTreeSimulator; last accessed October 14, 2016). HIV-1 subtype C sequences were simulated along these viral phylogenies using πBUSS (Bielejec et al. 2014), with substitution rates parameterized from analyses of African subtype C sequences. An overview of the simulation pipeline is shown in figure 1, and details about the parameter values and assumptions used in the DSPS-HIV and to generate phylogenies and sequences are found in supplementary table S2, Supplementary Material online. Notably, assumptions were made in sexual mixing partners, partner duration, interventions, sampling, and between- and within-evolution complexity. Disease progression and transmission within the DSPS-HIV are determined by set-point viral load using previously described relationships (Fraser et al. 2007). Simulations were parameterized to reflect estimates of prevalence and incidence from the peak of the HIV-1 epidemic in the late 1980s and early 1990s (Serwadda et al. 1992; Wawer et al. 1994), before treatment was widely available, with the root of the sequences dating back ∼40 years previously, coinciding with the recent subtype C estimates of a common ancestor in the 1940s (Faria et al. 2014). Further information about the DSPS-HIV will be available in a forthcoming publication.

### Regional Simulations

The Regional simulation model consists of a stochastic, individual-level epidemic transmission and intervention model, and an evolutionary model that generates viral phylogenies and sequence data to simulated transmission chains. Figure 1 and supplementary table S1, Supplementary Material online, describe the overall simulation pipeline, model components, parameters, and parameter values. Notably, assumptions were made on: sexual risk behavior (proportion of individuals in risk groups, mixing between risk groups, partner change rates); HIV infection (relative transmission rates); interventions (population-level effectiveness of ART); within-host evolution (neutral coalescent model, no co-infection and no recombination); between-host evolution (transmission of one virion, no recombination); and sequence sampling (at time of diagnosis of randomly selected individuals). To obtain the six epidemic scenarios shown in figure 2, we varied the relative transmission rate from early infections as well as parameters relating to uptake of the combination intervention respectively. The simulation algorithm is available from https://github.com/olli0601/PANGEA.HIV.sim (last accessed October 14, 2016), and combines (with further code): the individual-based HPTN071 (PopART) model version 1.1 to generate transmission chains, the VirusTreeSimulator (https://github.com/PangeaHIV/VirusTreeSimulator; last accessed October 14, 2016) to generate viral trees from transmission chains, and SeqGen version 1.3 (Rambaut and Grassly 1997) to simulate viral sequences along viral trees.

### Protocols for Phylogenetic Transmission Analyses

All participants adopted overall similar computational strategies that first reconstructed dated maximum-likelihood trees (Price et al. 2010; Stamatakis 2014; To et al. 2015), and then considered the viral trees fixed in one of the following transmission analyses:

#### ABC Kernel Method

Reporting variables were estimated with an experimental kernel-ABC method that combines a kernel method on tree shapes (Poon et al. 2013) with a framework for approximate Bayesian computation (ABC). The basic premise of ABC is that it is usually easier to simulate data from a model than to calculate its exact likelihood for the observed data. A model can then be fit to the observed data by adjusting its parameters until it yields simulations that resemble these data, bypassing the calculation of likelihoods altogether. We formulated a structured compartmental SI model (Jacquez et al. 1988) that was informed by the descriptions of the agent-based simulations that were distributed to all participants. Specifically, the model comprised three populations: a main local population, a second local high-risk minority population, and an external source population. Each population was further partitioned into susceptible and infected groups, where the latter was stratified into three stages of infection (acute, asymptomatic, and chronic). Mixing rates between the main and minority local populations were controlled by two parameters to allow for asymmetric mixing. Individuals with acute or asymptomatic infections migrated from the external region to the local region at a constant rate *m*, and replaced with new susceptible individuals in the external region. One infected individual in the external source population started the simulation. Coalescent trees were then simulated based on population trajectories derived from the numerical solution of the ordinary differential equations that represent the model, using the R package *rcolgem.* The subset tree kernel (Poon et al. 2013) was used as a distance measure between the simulated coalescent trees and the reconstructed viral phylogenies on available sequence data, or the provided phylogenies. A Markov chain Monte Carlo implementation of ABC was used to fit the model. This kernel-ABC approach was validated on simulated data from more conventional compartmental models (Poon 2015).

#### Birth–Death Skyline Method with Sampled Ancestors

Phylodynamic analyses were performed in BEAST v2.0 (Bouckaert et al. 2014) using the add-ons “bdsky” (Stadler et al. 2013), “SA” (Gavryushkina et al. 2014) and “bdmm” (Kühnert et al. 2016). Under the birth–death skyline model with sampled ancestors (“SA” module), individuals could transmit with some probability after sampling which improved estimation of the reporting variables in preliminary analyses (round 1 of the exercise). To estimate the proportion of early transmissions, the multi-type birth–death model was used with two compartments (“bdmm” module) to consider individuals in their first 3 months of infection separately from those in later stages of infection. In all analyses, time was partitioned into different intervals to obtain estimates of varying transmission rates through time. As further described in supplementary text S3, Supplementary Material online, for both Village and Regional simulations, lognormal priors were used for the effective reproductive number *(*mu = 0 and sigma = 0.75) and the becoming-non-infectious rate (lognormal with mu = −1 and sigma 0.5). Uniform priors were used for the sampling proportion, and specified based on available meta-data. For the Village datasets 0, 1, 2, 3, 4, 9, 10, 11 and 12, we assumed a priori a sampling proportion between 15% and 40%; for Village datasets 5, 6, 7 and 8 between 40% and 100%; and for the Regional datasets between 5% and 10%. The prior distribution for the removal probability *r* was chosen based on an estimate of the proportion of sampled infected individuals that are on treatment, and calculated from available survey data before intervention start. Sensitivity analyses on these prior choices were conducted. The reporting variables were estimated from MCMC output of the posterior model parameters using a customized procedure that is fully described in supplementary text S3, Supplementary Material online.

#### Bayesian Transmission Chain Analyser

The Bayesian approach reported in (Didelot et al. 2014) was adapted to account for incomplete sampling as well as heterogeneity in HIV transmission rates. In place of a susceptible-infectious-recovered (SIR) model (as in Didelot et al. 2014) a generalized branching model was used to describe transmission dynamics. In this model, the (prior) time interval between a case becoming infected and infecting others (*t*_gen_) is distributed such that there is a peak after infection, a chronic phase, and increased infectivity with progression to AIDS. Cases were sampled after a random time since becoming infected (*t*_samp_). The prior distribution of the numbers of secondary cases was negative binomial (*n* = 5, *P* = 0.7), reflecting a convolution of a Poisson distribution conditioned on a gamma-distributed overall infectivity. To account for infected individuals in transmission chains for whom a sequence was not available, likelihood terms were adjusted by numerically calculating the probability that a case infected at a given time had no sampled descendant cases by the time the study finished, and then conditioning on each case’s number of sampled and unsampled descendants. A reversible-jump Bayesian MCMC approach with proposal moves as described in (Didelot et al. 2014) was used to fit the model. This approach produces a posterior collection of transmission trees. From these, we extracted the portion of infections in the acute stage, recent changes in incidence and other outcomes required for the comparison study. The generation time *t*_gen_ had prior *t*_gen_ ∼ 0.4 gamma(1.3,1) + 0.6 gamma(3.5,3.5) where the arguments are the shape and scale parameters. The time to sampling had prior *t*_samp_ ∼ gamma(0.7, 1.5).

#### Structured Coalescent

Structured coalescent models were implemented in the rcolgem R package and were based on compartmental infectious disease models using the approach described in (Volz 2012). These models were tailored to the Regional and Village scenarios, and included compartments for stage of infection (early HIV infection through AIDS as in Cori et al. 2014), sex, and diagnosis/treatment status. Transmission rates were allowed to vary between compartments, and generalized logistic functions described secular trends in the force of infection through time. Coalescent models also included a deme for the unsampled source deme to capture the effects of lineage importation into the surveyed region. Models were fitted to the dated viral phylogenetic trees and to available epidemiological data under the approximation that the corresponding likelihood terms are independent. For the Regional simulations, the contribution to the likelihood model of the CD4 counts at diagnosis and gender of all sequenced individuals was assumed multinomial; the proportion of diagnoses with a sequence was assumed binomial; and that of survey data (sex, diagnosis, and treatment status) was assumed multinomial. For the Village simulations, fewer meta-data variables were available. The likelihood model assumed that estimated HIV prevalence was within the bounds given by the available survey data. A parallel Bayesian MCMC technique (Calderhead 2014) was used to obtain posterior distributions of model parameters.

### Statistical Analysis

Phylogenetic estimates and true values were transformed so that their differences were approximately normally distributed. For incidence and incidence reductions, the error $e_{i}\;$of response i was calculated as $e_{i}={\;\text{log}(\hat{x}}_{i})-\text{log}(x_{i})$, where ${\hat{x}}_{i}$ is the phylogenetic estimate and $x_{i}$ the true value on dataset i; for proportions, the error was calculated as $e_{i}={\hat{x}}_{i}-x_{i}$. Data points outside the whiskers of Tukey boxplots were considered as outliers.

To identify covariates associated with large error in phylogenetic estimates, stepwise model selection with the *stepGAIV.VR* procedure in the *gamlss* R package was used to reduce the number of covariates at significance level 0.01 (supplementary table S4, Supplementary Material online). The contribution of the remaining covariates to outlier presence (response) was evaluated with partial least squares (PLS) regression (Boulesteix and Strimmer 2007), because of the limited number of datasets and dependencies amongst the covariates. PLS regression is a dimension reduction technique that identifies combinations of covariates (PLS latent factors) that are maximally correlated with the response variable, and then regresses the response variable against the latent factors. The first four latent factors that explained most of the variance in outlier presence were considered in the error analysis. Figure 5B shows, in the notation of (Boulesteix and Strimmer 2007), the sign of the PLS regression coefficients $B_{j1}^{}$ for each covariate j to the univariate response variable across the first c=4 latent factors. The proportion of variance $p_{j}$ in the response variable attributable to each covariate j is calculated as $p_{j}=\sum_{k=1}^{c}(\frac{w_{jk}}{\left\|w\right\|}{)}^{2}\;v_{k}$, where $w_{jk}$ is the weight of covariate j to the kth latent factor and $v_{k}$ is the variance explained by the kth latent factor. PLS regression was performed with the *plsr* routine in the *pls* R package.

## Supplementary Material

Supplementary figures S1–S10, tables S1–S7, and text S1–S4 are available at *Molecular Biology and Evolution* online.

## Author Contributions

A.L.B. and C.F. conceived the study. O.R., E.H., A.L.B., and C.F. designed and coordinated the study. E.H., M.H., S.L., and A.L.B. designed and generated the Village simulations. M.H. contributed the virus tree simulator from transmission chains. M.P., A.C., O.R., and C.F. designed and generated the Regional simulations. O.R. checked the submissions received, performed the statistical analysis and wrote the first draft except parts of the “Methods” section. C.C., M.K., X.D., G.P., A.P., J.J., R.L., C.W., G.L., D.R., D.K., T.S., E.V., B.D., M.H., and S.F. evaluated the simulated data and wrote parts of the “Methods” section. All authors reviewed and approved the statistical analysis, and the final version of the article.

## Supplementary Material

Supplementary Data
