# Supplementary material for: Phylogenetic Tools for Generalized HIV-1 Epidemics: Findings from the PANGEA-HIV Methods Comparison
Source: Mol Biol Evol. 2016 Oct 7;34(1):185–203. doi: 10.1093/molbev/msw217 (PMC5854118; doi:10.1093/molbev/msw217)
Supplement: Supplementary Data [file msw217_suppl.zip › MBE-16-0805_OliverRatmann_v160928_Text_S4.pdf]

**Phylogenetic Tools For Generalized HIV-1 Epidemics: Findings from the PANGEA-HIV Methods Comparison**

**Supplementary Text S4: PANGEA-HIV specification of the birth-death skyline method with sampled ancestors**

We performed our phylodynamic analyses using the add-ons bdsky [2] and SA [3] in BEAST v2.0 [1]. We estimated the posterior distribution of the epidemiological parameters using fixed trees. In case of sequencing data, we first estimated time trees using RAXML/ExaML [4][6] & LSD [5], and then inferred epidemiological parameters in BEAST.

**Parameter inference**

For calculation of epidemiological parameters we assumed the birth-death skyline model [2] with sampled ancestors (SA) [3]. The model assumes a transmission rate lambda, removal rate without sampling $\mu$ and sampling rate $\Psi$. Upon sampling, an individual is removed with probability $r$ (called removal probability). It turned out to be crucial to allow for sampled individuals to further transmit with some probability (1-$r$)>0 (the original model in [2] assumed $r$=1).

Under the skyline model, time is partitioned into different intervals. Within each interval, the parameters are assumed to be constant. Across intervals, the rates may change in an arbitrary fashion.

For each interval, we estimate the effective reproductive number $R = \lambda / (\mu + \Psi r)$ and the becoming-non-infectious rate $\delta = \mu + \Psi r$ at which an infected individual becomes non-infectious. The transmission rate $\lambda$ is therefore given by $R*\delta$, and the sampling proportion is $\Psi /( \mu + \Psi)$.

*We partitioned time into three intervals:*

- The Village data has one interval over the evaluation period and two in the first 40 years, with their lengths being chosen such that there are an equal number of branching events in each interval. The data was not informative enough to split up the evaluation period into several intervals to gain a higher understanding of what happened during the treatment time. We assumed the HKY model for sequence evolution for the datasets consisting of sequences.
- For the Regional dataset, time was partitioned following the provided information. In particular, for the removal probability $r$ the first interval runs from 1970 until 2004, the second until 2015 and the third over the evaluation period, 2015-2019. For the sampling proportion $R$ and the becoming-non-infectious rate $\delta$, the intervals run from 1970 to 2000, the second until 2015 and the third over the evaluation period.

*Sampling proportion*
For the sampling proportion $\Psi / (\mu + \Psi)$, we set a prior according to the given sampling densities stated in the information for participants, i.e. for villages 0, 1, 2, 3, 4, 9, 10, 11 and 12 we assumed a uniform prior between 15 - 40% and for villages 5, 6, 7 and 8 between 40 – 100%. For the regional data we used a uniform prior for the sampling proportion between 5%-10%.

*Removal probability - Villages*
The prior distribution for the removal probability $r$ was chosen based on the probability of a sampled infected individual to be on treatment, and therefore presumed to be non-infectious. Using the provided numbers, for the villages, we calculated the ratio of number of patients on

treatment in year 40 ($T_{40}$, given in metadata) and the number of infected people at the end of year 39 ($P_{39}$*8000 with prevalence $P_{39}$ from metadata), $r = T_{40} / P_{39}*8000$. For village 0 the removal probability *r* was set 0, as no infected individuals are on treatment.

*Removal probability - Regionals*
For regionals, we defined *r* in the middle interval as the number of sampled individuals on treatment and sampled before 2015 out of the total number of samples. We defined *r* in the last interval as the number of sampled individuals on treatment and sampled after 2015 out of all samples.

*Prior assumptions for the epidemiological parameters R and δ*
We used a lognormal prior for *R* (lognormal with mu=0 and sigma=0.75) and $\delta$ (lognormal with mu=-1 and sigma 0.5) for the villeages and regions.

*Sensitivity analyses*
To explore sensitivity of our estimates towards prior assumptions, analyses were repeated with a wider prior on *R* and $\delta$ for villages (lognormal with mu=0.0 and sigma=1.5), as well as with removal probability $r$=0. To see how sensitive the calculation is to changes in the sampling proportion, the true sampling proportion (25% and 50% respectively) was used for an additional analysis. Results did not change noticeably using these stricter priors (not shown).

**Assessing criteria i-vi**
We used the posterior distributions for *R* and $\delta$ to assess the criteria i-vi of the simulation study.

Criteria i:
When analysing the Village data, we calculated the difference of expected incidence in year 44 ($EI_{44}$, see Criteria ii) and year 39 ($EI_{39}$, see Criteria ii). This difference was calculated for all values sampled by the MCMC, i.e. we obtained its posterior distribution. The 95% highest posterior density (HPD) interval was calculated to get the lower and upper bound for the difference.

The same calculation was performed on the Regional dataset, using the last year before the simulation ends (usually year 2018, except for Region F and O, where it is 2016) and year 2014.

If the HPD interval contained 0, we report no significant evidence rejecting stable incidence, if the HPD interval is entirely below 0 we report decreasing incidence, and if the HPD interval is above 0 we report increasing incidence.

Criteria ii:
We calculated incidence and number of susceptible people in year 43 based on our estimates of *R* and $\delta$ at the end of the evaluation period. We assumed that the total population at the end of year 43 is $N_{43}$ = 8000*1.01^4, based on a population size of 8000 at the end of year 39 and a growth rate of 1% per year. The number of infected individuals at end of year 43 is, $I_{43}$ = 8000* $P_{39}$* exp(($\lambda$ - $\delta$)*4), using $R = \lambda/\delta$ and $\delta$ estimates for the evaluation period and $P_{39}$ being the prevalence at end of year 39. Therefore the susceptible population size at the end of year 43 is $S_{43} = N_{43} - I_{43}$.

The expected incidence in year 44 ($EI_{44}$) was calculated by subtracting the number of infected individuals at the end of year 43 ($I_{43}$) from the number of infected individuals at the end of year 44 ($I_{44}$), and by adding the number of individuals (out of $I_{43}$) that became non-infectious

during year 44 ($BU_{44}$), i.e. $EI_{44} = I_{44}-I_{43}+BU_{44}$, with $BU_{44} = I_{43}$ (1- e^(- $\delta$)). The annual percent incidence was calculated via $\%INC_{t\_e} = EI_{44}/S_{43}$.

This value $EI_{44}/S_{43}$ was calculated for each sample of the MCMC, i.e. we obtained its posterior distribution. The final value was calculated by taking the mean of all $EI_{44}/S_{43}$ values. The 95% HPD interval was calculated to get the lower and upper bound $EI_{44}$ values.

Criteria iii:
Now we calculated the ratio *Ratio* = ($EI_{44}/S_{43}$) / ($EI_{39}/S_{38}$), with $N_{38}$ = 8000/1.01, and $I_{38}$ = 8000*$P_{39}$ * exp(-($\lambda$ - $\delta$)), where $\lambda$ and $\delta$ result from the middle interval in the skyline model. This value *Ratio* was calculated for all values sampled by the MCMC, i.e. we obtained its posterior distribution. The final value was calculated by taking the mean of all *Ratio* values. The 95% HPD interval was calculated to get the lower and upper bound *Ratio* values.

Criteria iv-vi
We addressed criteria iv-vi (proportion of transmissions that originated from individuals within their first 3 months of HIV infection) by employing the multi-type birth-death model [6]. This model allows us to analyse the data using exactly the same setup as described above, but with two different types of infected individuals: acute (within their first 3 months of HIV infection) and chronic individuals. This yields separate transmission rates $\lambda_a$ and $\lambda_c$ for transmissions caused by acute or chronic individuals, respectively, for each of the three intervals. We assume that $\delta_a = \delta_c$. All priors and interval lengths were set to the priors above, and the rate of becoming chronic was set so that individuals remained acutely infected for 3 months on average.The proportion of transmissions (criteria v & vi) that originated from acute individuals is *%Early* = ($f_a$ * $\lambda_a$)/ ($f_a$ * $\lambda_a$ + $f_c$ * $\lambda_c$), where $f_a$ and $f_c$ are the expected fractions of individuals in the acute and chronic state, respectively. Criteria iv was assessed if the mean of the posterior distribution for $P_a$ was below 10%, between 10-30%, or above 30%".

**Remarks**

A drawback of our approach is that we cannot directly infer incidence / prevalence, but only the epidemiological parameters (removal and effective reproductive number). From the parameters of the birth-death skyline model (i.e. R and $\delta$) we work out (in a slightly ad hoc way, as described above) the incidence values. Our model could be used to directly infer incidences using particle filtering approaches which remains to be investigated in future work.

**References**

[1] Bouckaert, R., Heled, J., Kühnert, D., Vaughan, T., Wu, C. H., Xie, D., ... & Drummond, A. J. (2014). BEAST 2: a software platform for Bayesian evolutionary analysis. *PLoS computational biology*, 10(4), e1003537.

[2] Stadler, T., Kühnert, D., Bonhoeffer, S., & Drummond, A. J. (2013). Birth–death skyline plot reveals temporal changes of epidemic spread in HIV and hepatitis C virus (HCV). *Proceedings of the National Academy of Sciences*, 110(1), 228-233.

[3] Gavryushkina, A., Welch, D., Stadler, T., & Drummond, A. J. (2014). Bayesian inference of sampled ancestor trees for epidemiology and fossil calibration. *PLoS computational biology*, *10*(12), e1003919.

[4] Stamatakis, A. (2014). RAxML Version 8: A tool for Phylogenetic Analysis and Post-Analysis of Large Phylogenies. *Bioinformatics*, 1;30(9):1312-3.

[5] T.-H. To, M. Jung, S. Lycett, O. Gascuel. Fast dating using least-squares criteria and algorithms. *Submitted*.

[6] Kühnert D, Stadler T, Vaughan TG, Drummand AJ. Phylodynamics with migration: A computational framework to quantify population structure from genomic data. *Under review*.
