# Supplementary material for: Phylogenetic Tools for Generalized HIV-1 Epidemics: Findings from the PANGEA-HIV Methods Comparison
Source: Mol Biol Evol. 2016 Oct 7;34(1):185–203. doi: 10.1093/molbev/msw217 (PMC5854118; doi:10.1093/molbev/msw217)
Supplement: Supplementary Data [file msw217_suppl.zip › MBE-16-0805_OliverRatmann_v160928_Text_S1.pdf]

Phylogenetic Tools For Generalized HIV-1 Epidemics: Findings from the PANGEA-HIV Methods Comparison

Supplementary Text S1: Supplementary tables and figures

Table S1 Model components and assumptions of the Regional Model.

Model parameters	Choice of parameter values	Type of evidence
**Demographics**<br>Individuals enter the modelled population continuously at age 13, leaving the population when they die, either from disease related mortality or other reasons. The overall population is slowly growing in size at a rate similar to South Africa.		
Population size in 1960	15,000 individuals aged 13 or older	Assumed
Number of new individuals aged 13 per year	The fertility rate was age-dependent, and calibrated to UNPD WPP 2006 South Africa estimates. A truncated Gamma distribution with shape parameter 15, scale parameter 1.6, minimum 13 years and maximum 50 years was used. At any time step in the model simulation, the number of new individuals of age 13 was obtained as the expected number of new arrivals under the truncated Gamma distribution. New individuals had 50% probability of being male.	Empirical
Population mortality rate	The mortality rate was age- and time-dependent, and calibrated to World Bank Global Health Observatory data (http://apps.who.int/gho/data/view.main.1360?lang=en). The final model had the form $\exp(0.001*(a\text{-}13)^{1.7} + 0.002*(a\text{-}13)^{1.7} * 60/(t\text{-}1900) - 4.9)$.	Empirical
**Sexual partnerships**<br>Once individuals entered the model, they may form and break up sexual partnerships. Individuals form partnerships assortatively by risk group and age. All partnerships are heterosexual.		
Sexual risk behavior	3 risk groups categories (high/medium/low) as in (1).	Assumed
Proportion in high/ medium/ low risk group when entering population	Men: 50% / 40% / 10%; Women: 60% / 30% / 10%.	Assumed
Maximum number of simultaneous partners	High: 10; Medium: 3; Low: 1.	Assumed
Rate of partner acquisition	1.2 partners per year if maximum number of simultaneous partners not reached.	Assumed
Mixing between risk groups	10% of one's partnerships made within a risk group (assortatively), and the remainder made at random in any group.	Assumed
Mixing between age groups	Strongly assortative, determined from Manicaland cohort survey data.	Empirical
Partnership duration	Gamma distribution with shape parameter 10 and scale parameter 2.5.	Assumed
**Viral Introductions**<br>The epidemic was seeded in 1980 and further viral introductions occurred throughout the simulation.		
Seed cases	The simulation was initially run for 20 years without HIV-1, to allow partnerships to reach a steady state. In 1980, 0.5% of low-risk and 1% of medium and high-risk individuals are seeded HIV-1 positive, with HIV-1 transmission occurring from that point onwards.	Assumed
Proportion of viral introductions among annual	5% or 20%, range includes frequent viral introductions as reported in settings with highly mobile populations (2, 3)	Varied in simulations

new cases		
**HIV-1 infection**<br>HIV-1 negative individuals are exposed to risk of infection when they are in a serodiscordant partnership. Infection can occur at any time during a serodiscordant partnership, with the risk of infection depending upon the HIV-1 stage of the infected partner, and whether they are on ART or not at that time. For male HIV-1 negative individuals risk of infection also depends on their circumcision status.		
Proportion in SPVL group < 4, 4-4.5, 4.5-5, ≥ 5 $\log_{10}$ copies/μL after seroconversion	25% in each group, similar to (4).	Empirical
Duration of early transmission phase (months)	Sampled uniformly between 1 to 5 months, from (5).	Empirical
Duration of CD4 stages >500, 350-500, 200-350 and ≤200 cells/mm^3 when not on ART	Sampled uniformly from ranges in (4) dependent on CD4 stage and SPVL group.	Empirical
Duration CD4 stages >500, 350-500, 200-350 and ≤200 cells/mm^3 when on ART	No progression if virally suppressed; duration of each CD4 stage is doubled if virally unsuppressed as in (1).	Assumed
CD4 of individual after end of early transmission phase	Individuals can start at a lower CD4 stage with a probability from (4) dependent on their SPVL group.	Empirical
Probability of transmission from individual with CD4>500cells/mm^3 not on ART, per time step	Baseline transmission probability	Calibrated to model incidence and prevalence
Relative increase in transmission probability during early transmission phase	6.0 (when ~10% of transmissions early) and 26.0 (when ~40% of transmissions early), values from (5, 6)	Varied in simulations
Relative increase in transmission probability (compared to baseline transmission probability) when CD4 350-500 / 200-350 / ≤200 cells/mm^3	1.0 / 1.9 / 3.0	Assumed
**Intervention**<br>The intervention model includes HIV-1 testing, male circumcision, ART provision and loss-to-follow up from ART.<br><br>HIV-1 testing is divided into two separate rates. Firstly, there is a standard of care (background) rate that increases over time, with HIV-1 testing beginning in 2000 and ART becoming available in 2004, reflecting historical scale-up of testing in sub-Saharan Africa. Secondly, starting in 2015, intensive annual testing rounds are modelled, that mimic the HIV-1 testing component of the HPTN-071 combination HIV-1 prevention intervention.<br><br>Men testing HIV-1 negative, who were not previously circumcised, are offered medical male circumcision in the model. Once circumcised, susceptibility to HIV-1 is reduced. Medical male circumcision rates differ over time, reflecting historical scale-up and strengthened testing as part of the combination prevention intervention.<br><br>Individuals only start ART after a positive HIV-1 test result, although they may be lost to follow-up before this occurs. After ART start, individuals remain virally unsuppressed during an early ART period. This periods lasts on average 6 months. Thereafter, individuals become either virally suppressed, or not fully suppressed. Infectivity is reduced when an individual is on ART, but it is more substantially reduced if individuals are virally suppressed. Individuals on ART may drop out of treatment at any time after ART start. After drop-out, individuals may re-start therapy. The proportion of HIV+ individuals on ART under the different intervention scenarios is shown in figure 2 of the main text.		
Relative reduction in	0.6, from (7-9)	Empirical

susceptibility when circumcised		
Effectiveness of ART when no virally suppressed, or during early ART	0.45	Assumed
Effectiveness of ART when virally suppressed	0.9	Assumed
Annual intervention coverage	Fast: 90%; Slow: 20%; No intervention: 0%.	Varied in simulations
Uptake (% who successfully start ART)	Background: 30% (CD4>200); 60% (CD4≤200 cells/mm^3)<br>Intervention: 50% (CD4>200); 75% (CD4≤200 cells/mm^3)	Assumed

Sequence Sampling

Since 2000, sequences were randomly sampled at time of diagnosis in proportion to the number of annual new diagnoses. The proportions of individuals sampled between 2000-2014 and 2015-2020 are controlled by two parameters. One sequence was sampled per individual. The first parameter is the total number of sequences sampled. The second parameter is the proportion of sampled sequences that are obtained after intervention start in 2015. In addition, the sampling duration was also varied.

Duration of sampling after intervention start.	3 years or 5 years.	Varied in simulations
Total number of sequences sampled	1600 or 3600, corresponding to 8% and 16% sequence coverage in the last year of the simulation. In comparison to the large sequence data sets that are available for concentrated epidemics in Europe or North America, these lower values reflect challenges in achieving high sequence coverage where large populations are infected.	Varied in simulations
Proportion of sampled sequences that are obtained after intervention start in 2015.	50% or 85%, corresponding to strong increases in sequence coverage after intervention start as expected in trial settings (10-12).	Varied in simulations

Ancestral relationships of HIV-1 viruses

The topology of viral phylogenies does not necessarily correspond to the transmission tree, especially when viral infections persist life-long (13). To allow for such disagreement, we used a particular within- and between host coalescent model that is more fully described elsewhere (14, 15). The same model was used in the Village simulations.

For each transmission chain, viral phylogenies with branch lengths in calendar time are generated through recursive application of a neutral within-host coalescent model. The infection time of the index case is considered as root of the within-host phylogeny of the index case, and any onward transmission events or sampling events as tips. Under these tip and date constrains, the within-host phylogeny of the index case is simulated assuming an increasing effective population size. For each new infection, the process is repeated and the within-host phylogenies of newly infected individuals are concatenated to the corresponding transmission tips of their transmitter. The model assumes that a single transmitted virion leads to clinical infection of the newly infected individual. For each transmission chain, the simulation produces a dated viral phylogeny that is rooted at the index case and has as tips the sampling times of all individuals in the same transmission chain that are sampled.

The sub-trees that correspond to each transmission chain were concatenated to one multi-furcating root in order to obtain a single tree. For each sub-tree, the branch length of each sub-tree to the root reflects the time between the root age and the time of infection of the index case of the corresponding transmission chain in the model population.

Within-host population size model	The logistic effective population size model is inherited from BEAST, BEAST::LogisticGrowthN0, with parameters N0tau=1, r=2.851904, v.T50=-2. These parameters were chosen so the final effective population size is broadly similar to estimates typically obtained with a BEAST Skyline model (16). See figure S11.	Assumed
Transmission bottleneck size	One virion transmitted.	Assumed

Age of multi-furcating root	Set so that the root age corresponds to estimated dates of origin of subtype C virus in South Africa (17).	Empirical
**Sequence evolution**<br>Viral sequences were simulated along the viral tree from a starting sequence.<br><br>To this end, branch lengths of the tree were first translated from calendar time to average number of substitutions per site per year. The evolutionary rate model included two components to reflect differences in evolutionary rates along transmission and non-transmission lineages (18, 19). Because one sequence is sampled per individual under the Regional model, non-transmission lineages correspond to the part of tip branches that correspond to viral evolution within sampled individuals. All other branches are part of transmission lineages. Evolutionary rates were drawn from two rate models of transmission and non-transmission lineages, and multiplied with branch lengths in units of calendar time to obtain branch lengths in units of average number of nucleotide substitutions per site per year.<br><br>The starting sequence, from which all viral sequences were simulated, was obtained through ancestral state reconstruction of full-genome HIV-1 subtype C sequences.<br><br>Viral sequences were simulated for the *gag*, *pol* and *env* genes from the starting sequence under a codon-based GTR+G sequence evolution model for each gene. The simulated *gag* gene was 1440 nucleotides long, the *pol* gene 2844 nucleotides, and the *env* gene 2523 nucleotides.		
Evolutionary rate of transmission lineages	Sampled from lognormal density with mean evolutionary rate 0.0022 and standard deviation (on the log scale) 0.3. Parameterized from phylogenetic analyses of subtype C sequences from southern Africa. See figure S12.	Empirical
Evolutionary rate of non-transmission lineages	Sampled from lognormal density with mean evolutionary rate 0.0044 and standard deviation (on the log scale) 0.5. Set to twice the rate of transmission lineages (18, 19). See figure S12.	Assumed
Nucleotide substitution rates	Informed from phylogenetic analyses of subtype C sequences from southern Africa. See figure S13.	Empirical

Table S2 Model components and assumptions of the Village Model.

Model parameters	Choice of parameter values	Type of evidence
**Demographics**<br>Age is not explicitly modelled. Individuals enter the modelled population at 'birth,' already sexually mature, and leave the population when they die, either from disease related mortality or other reasons. The overall population is slowly growing in size.		
Population growth	Population growth was set at 1%/year to achieve incidence/prevalence comparable to a small Ugandan fishing village (20, 21).	Calibrated
**Sexual partnerships**<br>Individuals are in a sexual partnership with one other individual. Partnerships are formed at 'birth' and last until the death of either partner. No partner switching is modelled. Individuals form partnerships assortatively based on risk group, and the frequency of extra-partner contacts is also determined by risk group. Sex workers do not form partnerships.		
Sexual risk behavior	3 risk group categories	Assumed
Proportion in risk group when entering population	Men: High: 50% / Low: 50%; Women: High: 47% / Low: 47% / Sex Worker: 6%.	Assumed
Mixing between risk groups	All partnerships are within the same risk group. 50-80% of contacts are with partner (if present); remaining contacts are weighted by risk group (ex: high risk more likely to contact other high risk and to contact sex workers)	Assumed

Partnership duration	Partnership lasts until the death of a partner	Assumed
**Viral Introductions**<br>The epidemic begins with 1 infection in year 0. In simulations where imported sequences were included, this initial infection is also the ancestor of the 'imported' sequences. Viral introductions from outside of the focal population occur stochastically throughout the simulation.		
Seed cases	One female sex worker is infected at year 0, who automatically infects the populations outside the focal population, where the strain can evolve independently. HIV-1 transmission occurs from this point onwards.	Assumed
Proportion of viral introductions	Half of the simulations had no imported sequence migration. In simulations where this was included, 20% of transmissions were descendants of imported sequences by the end of the simulation.	Varied in simulations
**HIV-1 infection**<br>HIV-1 negative individuals are exposed to risk of infection when they make a serodiscordant contact, either with their partner or with another individual. Transmission risk is dictated by stage of infection and set-point viral load, with acute stage and higher viral loads conferring higher transmission risk. If the HIV-1 infected individual is on ART, transmission does not occur. Individuals also only become infectious 2 weeks after infection.		
Set-Point Viral Load Value	4.5 $\log_{10}$ copies/mL, based on mean of subtype C infected individuals in the UK HIV epidemic	Empirical
Duration of early transmission phase (months)	3 months	Assumed
Probability of transmission	Values based on equations given in (22), based on viral load and whether in acute stage. Divided by 100 to convert to per-act rather than per-year risks.	Empirical
Relative increase in transmission probability during early transmission phase	0 (when ~4% of transmissions early) and 12.5 (when ~20% of transmissions early), value from (22).	Varied in simulations
**Intervention**<br>No intervention or treatment is available before year 40. After year 40, ART is provided to approximately 20% of the population, including all sex workers. All individuals on ART are immediately fully suppressed, with viral load reduced to 50 copies/mL. ART is permanent (there is no loss to follow-up). As viral load determines disease progression, these individuals live much longer than individuals not on ART.		
Relative reduction in susceptibility when on ART	0.005 from (22).	Empirical
Relative increase in ART uptake in the 'fast' ART simulations	1.49	Assumed
**Sequence Sampling**<br>Since year 40, sequences were randomly sampled at some point after acute infection. Sampling was done after the simulation was complete, with either 25% or 50% of the total number of individuals HIV+ at any time between years 40 and 45, with approximately the same number of individuals sampled each year.<br>In simulations that were released as sequences only, 42 individuals were also sampled from the pre-intervention time period to replicate limited availability of older samples and to aid in phylogenetic reconstruction.		
Duration of sampling after intervention start.	3 years or 5 years.	Varied in simulations
Total number of sequences sampled	Between 638 and 1996, corresponding to 25% and 50% of the total number of infected individuals in the last 5 years of the simulation. This represented a situation where a small population was intensively sampled for the duration of an intervention, simulating a 'best possible' scenario where high sequence	Varied in simulations

	coverage was available.	
Proportion of sampled sequences that are obtained after intervention start in year 40.	100% in all simulations released as phylogenies. 95% in the four simulations released as sequences, corresponding to strong increases in sequence coverage after intervention start as expected in trial settings (10-12).	Varied in simulations
**Ancestral relationships of HIV-1 viruses**<br>The topology of viral phylogenies does not necessarily correspond to the transmission tree, especially when viral infections persist life-long (13). To allow for such disagreement, we used a particular within- and between host coalescent model that is more fully described elsewhere (14, 15). The same model was used in the Regional simulations.<br><br>For the transmission chain, viral phylogenies with branch lengths in calendar time are generated through recursive application of a neutral within-host coalescent model. The infection time of the index case is considered as root of the within-host phylogeny of the index case, and any onward transmission events or sampling events as tips. Under these tip and date constrains, the within-host phylogeny of the index case is simulated assuming an increasing effective population size. For each new infection, the process is repeated and the within-host phylogenies of newly infected individuals are concatenated to the corresponding transmission tips of their transmitter. The model assumes that a single transmitted virion leads to clinical infection of the newly infected individual. For the transmission chain, the simulation produces a dated viral phylogeny that is rooted at the index case and has as tips the sampling times of all individuals that are sampled.<br><br>As all transmissions in the simulation descend from a single ancestral infection, there is only one transmission chain, and all generated sequences naturally coalesce to one ancestral sequence.		
Within-host population size model	The logistic effective population size model is inherited from BEAST, BEAST::LogisticGrowthN0, with parameters N0tau=0.00593, r=2.851904, v.T50=-2. See figure S11.	Assumed
Transmission bottleneck size	One virion transmitted.	Assumed
Age of multi-furcating root	Set so that the root age corresponds to estimated dates of origin of subtype C virus in South Africa (17).	Empirical
**Sequence evolution**<br>Viral sequences were simulated along the viral tree from a starting sequence, which was obtained through ancestral state reconstruction of full-genome HIV-1 subtype C sequences from southern Africa by Gonzalo Yebra.<br><br>Each viral phylogeny was run through piBUSS three times, once each for *gag*, *pol*, and *env*. All parameters used to simulate the sequences were taken from BEAST analysis of full-genome HIV-1 subtype C sequences from Southern Africa.<br><br>Viral sequences were simulated for the *gag*, *pol* and *env* genes from the starting sequence under a codon-based GTR+G sequence evolution model for each gene. The simulated *gag* gene was 1479 nucleotides long, the *pol* gene 2999 nucleotides, and the *env* gene 2507 nucleotides.		
Nucleotide substitution rate gamma distribution shape parameter	Codons 1&2: 7.743; codon 3: 11.688	Empirical
Nucleotide substitution rate - *env*	Sampled from gamma distribution with mean evolutionary rate for codons 1&2: 2.98E-3 and codon 3: 5.52E-3, both with standard deviation 9.49E-7	Empirical
Nucleotide substitution rate – *gag*/*pol*	Sampled from gamma distribution with mean evolutionary rate for codons 1&2: 1.49E-3 and codon 3: 2.76E-3, both with standard deviation 4.75E-7	Empirical
Transition/transversion ratio	Codons 1&2: 0.139; codon 3: 0.765	Empirical

Table S3 Responses to the Phylodynamic Methods Comparison Exercise

Simulation model	Data set	Responses				
		Team Cambridge	Team Cambridge-London	Team Basel-Zürich[§]	Team London	Team Vancouver[§]
		(Total responses to the 5 reporting variables for each data set)				
**Regional**						
	D	0	0	0	5	5 [*]
	C	0	0	0	5	5 [*]
	A	0	0	0	5	5 [*]
	B	0	0	0	5	5 [*]
	O	0	5	5	5	0
	T	0	5	5	5	0
	S	0	5	5	5	0
	I	0	5	5	5	0
	R	0	4	5	5	0
	Q	0	5	5	0	0
	G	0	5	5	5	0
	N	0	5	5	5	0
	F	0	5	5	5	0
	L	0	5	5	5	0
	J	0	5	5	5	0
	P	0	5	5	0	0
	H	0	5	5	5	0
	K	0	4	5	5	0
	E	0	5	5	0	0
	M	0	5	5	5	0
**Village**						
	3	3	5	5	5	5
	2	3	5	5	5	5
	1	3	5	5	5	5
	4	3	5	5	5	5
	5	0	5	5	5	5
	11	0	5	5	5	5
	8	0	5	5	5	5
	9	0	5	5	5	5
	0	0	0	5	5	5
	6	0	5	5	5	5
	12	0	5	5	5	2
	7	0	5	5	5	5
	10	0	5	5	5	5

[§] Teams Basel-Zürich and Vancouver updated %Incidence estimates (Primary objective 2) after the data sets were unblinded. [*] Where sequences were provided, participants used full viral genomes (*gag+pol+env)* for inference. Team Vancouver also provided estimates based on partial *pol* sequences for two reporting variables on the indicated data sets.

Table S4. Estimating incidence and incidence reduction after a community-based intervention with phylogenetic methods on simulated PANGEA data sets.

Statistic	Responses

	Team Vancouver	Team Cambridge	Team Cambridge-London	Team Basel-Zürich	Team London
**Correlation between phylogenetic estimates and true values**					
%Incidence [1]	0.15	-0.78	0.91	0.83	0.64
Incidence ratio [2]	0.66	0.10	0.92	-0.07	0.15
**Bias**					
**(Overall)**					
%Incidence [3]	7.90	-1.83	0.35	3.15	0.57
Incidence ratio [3]	0.36	0.38	0.10	0.17	0.19
**(Village Simulation Model)**					
%Incidence [3]	7.75	-1.83	0.30	7.20	0.06
Incidence ratio [3]	0.50	0.38	0.11	0.21	0.13
**(Regional Simulation Model)**					
%Incidence [3]	8.31	-	0.39	0.44	1.06
Incidence ratio [3]	-0.06	-	0.09	0.12	0.23
**Mean absolute error on the log scale for cross-comparison**					
**(Overall)**					
%Incidence [4]	1.28	0.83	0.25	0.97	0.56
Incidence ratio [4]	0.43	0.39	0.14	0.32	0.33
**(Village Simulation Model)**					
%Incidence [4]	1.10	0.83	0.21	1.02	0.37
Incidence ratio [4]	0.50	0.39	0.14	0.26	0.20
**(Regional Simulation Model)**					
%Incidence [4]	2.07	-	0.29	0.98	0.71
Incidence ratio [4]	0.23	-	0.14	0.37	0.42

[1] Denote true % HIV-1 incidence per year after the intervention in PANGEA data set i by h_i, and estimated incidence by $\hat{h}_i$. Outliers with $\hat{h}_i$>20% were excluded, and the sample Pearson correlation between the remaining $\hat{h}_i, h_i$ is reported. [2] Denote true incidence ratios after the intervention in PANGEA data set i by r_i, and estimated incidence ratios by $\hat{r}_i$. Outliers with $\hat{r}_i$>2 were excluded, and the sample Pearson correlation is reported. [3] Bias estimates of incidence and incidence reduction was calculated as $1/n \sum_i \hat{h}_i - h_i$ and $1/n \sum_i \hat{r}_i - r_i$ respectively, after outliers were removed as described above. [4] Mean absolute error in phylogenetic estimates of incidence and incidence reductions was calculated as $1/n \sum_i |log\ \hat{h}_i - log\ h_i|$ and $1/n \sum_i |log\ \hat{r}_i - log\ r_i|$ respectively on the log scale for cross-comparison, after outliers were removed as described above.

Table S5. Identification of HIV-1 incidence trends during a community-based intervention with phylogenetic methods on simulated PANGEA data sets.

True incidence trend	Classified as	Responses				
		**Team Vancouver**	**Team Cambridge**	**Team Cambridge-London**	**Team Basel-Zürich**	**Team London**
		Number (Percentage of responses correctly classified)				
Larger than 25% reduction in incidence	Declining	4 (44%)	0 (0%)	15 (88%)	9 (47%)	11 (55%)
	Stable	4	1	2	4	9
	Increasing	1	1	0	6	0
	Scenarios not evaluated	14	21	6	4	3
No or smaller than 25% reduction in incidence	Declining	0	1	1	3	4
	Stable	3	0	5	3	2
	Increasing	2	0	0	1	1
	Scenarios not evaluated	2	6	1	0	0

Table S6. Estimating the proportion of early transmissions before and after a community-based intervention with phylogenetic methods on simulated PANGEA data sets.

Statistic	Responses			
	**Team Vancouver**	**Team Cambridge-London**	**Team Basel-Zürich**	**Team London**
**Correlation between phylogenetic estimates and true values (Village Simulation Model)** [1]				
Just before the intervention	0.46	0.69	0.69	0
After the intervention	0.59	0.83	0.28	0
**Correlation (Regional Simulation Model)** [1]				
Just before the intervention	0.72	0.90	0.20	0.13
After the intervention	0.53	0.92	0.49	0.71
**Bias (Village Simulation Model)** [2]				
Just before the intervention	3.9	11.8	1.4	-2.7
After the intervention	2.7	10.1	-2.6	-5.3
**Bias (Regional Simulation Model)** [2]				
Just before the intervention	-13.3	-2.1	-9.4	-19.6
After the intervention	-11.9	-1.4	-13.2	-17.2
**Mean absolute error (Village Simulation Model)** [3]				
Just before the intervention	7.3	12.0	5.3	7.0
After the intervention	6.2	10.1	6.6	6.8
**Mean absolute error (Regional Simulation Model)** [3]				
Just before the intervention	13.3	4.1	14.8	20.0
After the intervention	12.0	3.9	13.2	18.0

[1] Denote true % early transmission just before or after the intervention in PANGEA data set i by p_i, and estimated proportions by $\hat{p}_i$. The sample Pearson correlation is reported. [2] Bias was calculated as $1/n \sum_i \hat{p}_i - p_i$. [3] The mean absolute error was calculated as $1/n \sum_i |\hat{p}_i - p_i|$.

Table S7. Significant predictors of error in phylogenetic estimates on simulated PANGEA data sets.

Primary objective	Covariates varied in the simulations (values of covariates) [1]	Significance of association with error			
		Team Cambridge-London	Team Basel-Zürich	Team London	Team Vancouver
		(P-value significance codes: ***: p<1e-3; **: p= 0.001-0.01; - : p>0.05)			
**Incidence after intervention**	True incidence after intervention (numerical)	***	-	0.02	-
	Simulation model (Village or Regional)	**	***	***	0.01
	Data provided (Sequences or trees)	-	-	-	-
	Frequency of viral introductions (<=5% or 20%)	-	***	-	-
	Sampling coverage at end of simulation (standard or high [2])	0.01	0.02	***	-
	Sampling duration after intervention start (3 years or 5 years)	0.04	-	-	-
	Proportion of sequences from after intervention start (50% or >80%)	-	***	0.04	-
**Incidence reduction during intervention**	True incidence ratio (numerical)	-	***	***	**
	Simulation model (Village or Regional)	-	***	0.03	0.02
	Data provided (Sequences or trees)	-	0.02	-	-
	Frequency of viral introductions (<=5% or 20%)	-	***	-	-
	Sampling coverage at end of simulation (standard or high [2])	-	-	-	-
	Sampling duration after intervention start (3 years or 5 years)	0.02	-	-	-
	Proportion of sequences from after intervention start (50% or >80%)	-	-	**	-
**Proportion of early transmissions just before intervention**	True proportion of early transmissions just before intervention (numerical)	0.01	***	***	**
	Simulation model (Village or Regional)	**	-	-	-
	Data provided (Sequences or trees)	0.017	-	0.03	-
	Frequency of viral introductions (<=5% or 20%)	-	0.02	***	-
	Sampling coverage at end of simulation (standard or high [2])	-	-	-	0.04
	Sampling duration after intervention start (3 years or 5 years)	-	0.04	-	-

	Proportion of sequences from after intervention start (50% or >80%)	-	0.02	-	-
**Proportion of early transmissions after intervention**	True proportion of early transmissions after intervention (numerical)	-	**	***	***
	Simulation model (Village or Regional)	**	-	-	-
	Data provided (Sequences or trees)	0.036	-	-	-
	Frequency of viral introductions (<=5% or 20%)	-	-	-	-
	Sampling coverage at end of simulation (numerical)	-	-	-	-
	Sampling duration after intervention start (3 years or 5 years)	-	-	-	-
	Proportion of sequences from after intervention start (50% or >80%)	-	-	-	-

[1] For each objective, the error e_i in phylogenetic estimates was defined so that errors were approximately normally distributed. Specifically, $e_i = log(\hat{x}_i) - log(x_i)$ for estimates $\hat{x}_i$ and true values x_i of incidence and incidence reduction, and $e_i = \hat{p}_i - p_i$ for estimates $\hat{p}_i$ and true values p_i of proportion of early transmissions. As predictors, we considered all variables v_j along which the PANGEA data sets were systematically varied in table 3. Variables took on either numerical or categorical values as indicated in brackets. We identified those v_j that were significantly associated with e_i. Specifically, we started with the full regression model containing all v_j as explanatory variables and then sequentially dropped predictors according to the generalised likelihood ratio test based on the BIC criterion (function stepGAIV.VR in the gamlss R package with $k = log(n)$).
[2] 16% versus 8% in the Regional data sets and 50% versus 25% in the Village data sets.

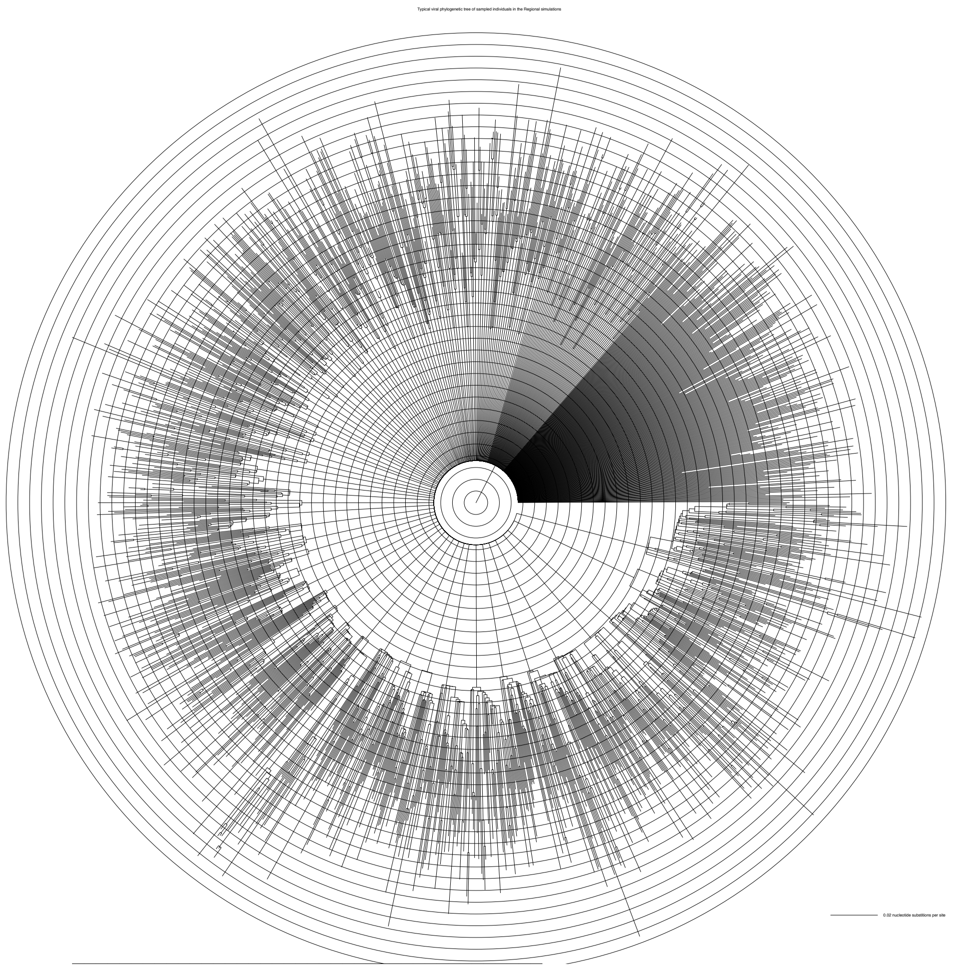

Figure S1. Viral tree of sampled individuals in a typical Regional simulation. 1600 (8%) of infected individuals from transmission chains in the Regional population were usually sampled. Due to frequent viral introductions, a large number of separate transmission chains were present in the modelled population. Each transmission chain was collapsed to sampled individuals. Corresponding viral trees with branch lengths in units of average nucleotide substitutions per site were generated under a coalescent model that also accounted for within-host viral evolution. Viral trees were connected to a single root sequence, with the root branch lengths reflecting time of viral introduction. The resulting tree of a typical Regional simulation is shown. Viral sequences were simulated along this viral tree.

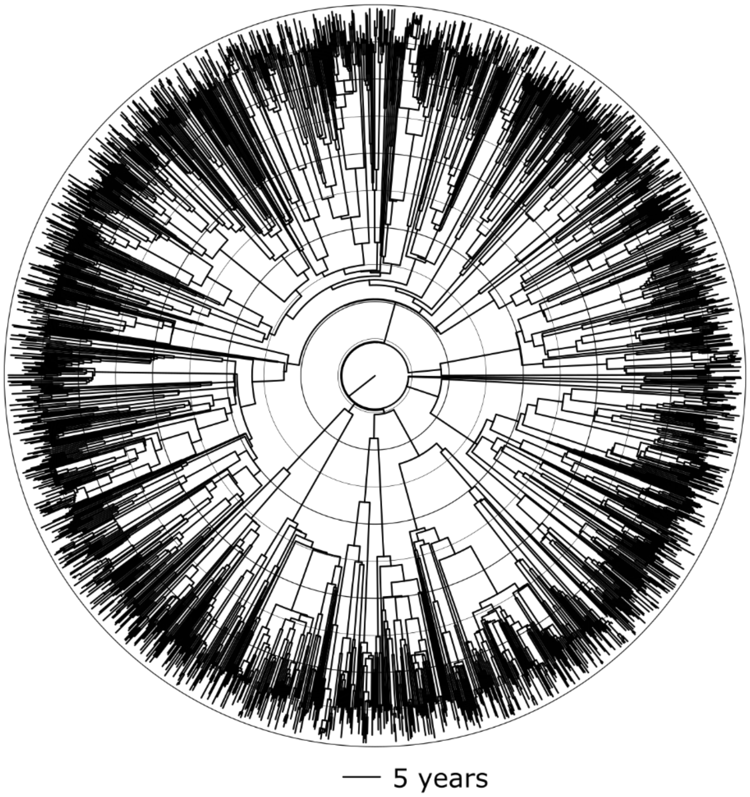

Figure S2. Viral tree of sampled individuals in a typical Village simulation. 638 to 1996 (25%-50%) of infected individuals from the entire transmission chain in the Village population were usually sampled. Parts of the entire transmission chain were modelled to have taken place outside the Village population. The transmission chain was collapsed to sampled individuals. Corresponding viral trees with branch lengths in units of time were generated under a coalescent model that also accounted for within-host viral evolution. The resulting tree of a typical Village simulation is shown. Viral sequences were simulated along this viral tree.

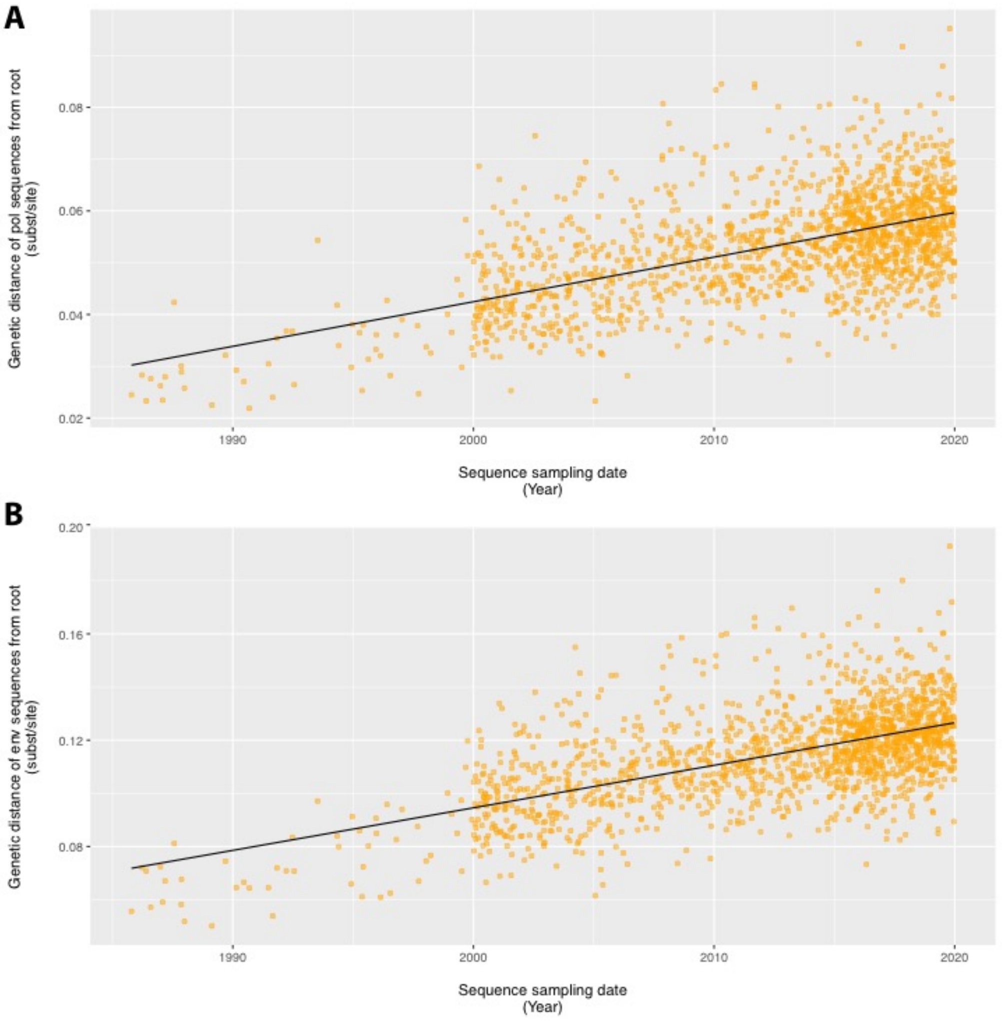

Figure S3. Root to tip divergence of a maximum likelihood trees reconstructed from viral sequences generated under the Regional model. One indicator of realism of simulated HIV-1 sequences is the degree to which viral evolution can be described by a single molecular clock. Viral trees were reconstructed with maximum-likelihood methods from simulated HIV-1 *pol* and *env* genes, and the patristic distances between the root and sampled taxa were plotted against sequence sampling dates. A linear regression model was fitted. (A) For *pol,* the mean evolutionary rate was 0.9e-3 subst/site/year. The variance explained by the constant clock model was $R^2 = 31\%$. (B) For *env,* the mean evolutionary rate was 1.6e-3 subst/site/year. The variance explained by the constant clock model was $R^2 = 35\%$.

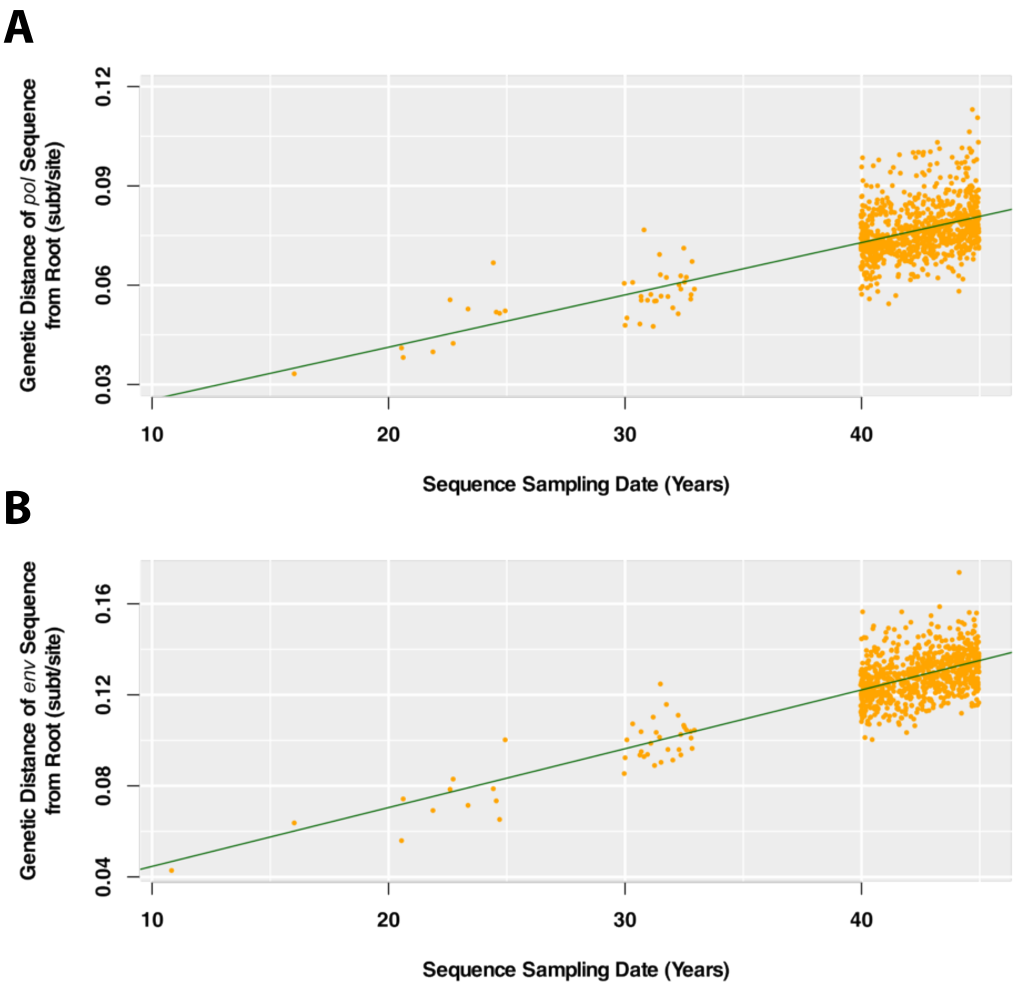

Figure S4. Root to tip divergence of a maximum likelihood trees reconstructed from viral sequences generated under the Village model. One indicator of realism of simulated HIV-1 sequences is the degree to which viral evolution can be described by a single molecular clock. Viral trees were reconstructed with maximum-likelihood methods from simulated HIV-1 *pol* and *env* genes, and the patristic distances between the root and sampled taxa were plotted against sequence sampling dates. A linear regression model was fitted. (A) For *pol,* the mean evolutionary rate was 1.6e-3 subst/site/year. The variance explained by the constant clock model was $R^2 = 34\%$. (B) For *env,* the mean evolutionary rate was 2.6e-3 subst/site/year. The variance explained by the constant clock model was $R^2 = 52\%$.

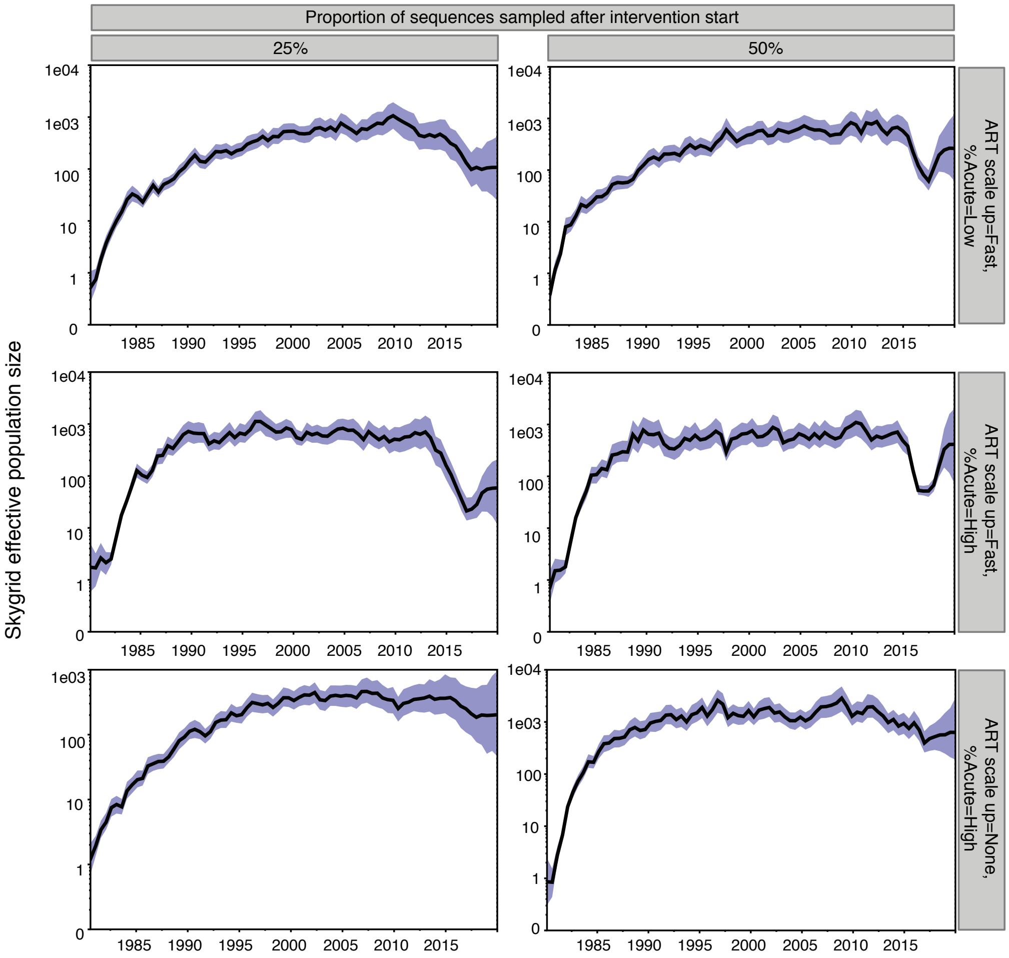

Figure S5. Signal to noise indicators for estimating incidence reductions on the Regional simulations at 8% sequence coverage. Skylines plots were reconstructed under the BEAST 1.8 Skygrid model to indicate if sufficient signal is present in the simulations to identify changes in incidence towards the present. For this analysis, true viral trees as provided for data sets E-T were used, using a previous multi-locus approach (23). Skyline plots are shown for data sets L, M, T (right column), and data sets similar to L, M, T but time homogeneous sequence sampling (~75 sequences sampled per year since 2000) (left column). For data sets L and T, incidence fell by approximately 60% reduction during the intervention. For data set M, incidence declined by approximately 10% as a result of improving standard of care. Qualitatively, these differences are visible in the Skyline plots under time homogeneous sequence sampling (left column). However, under rapidly increasing sampling as in the released data sets, these differences appear confounded (right column).

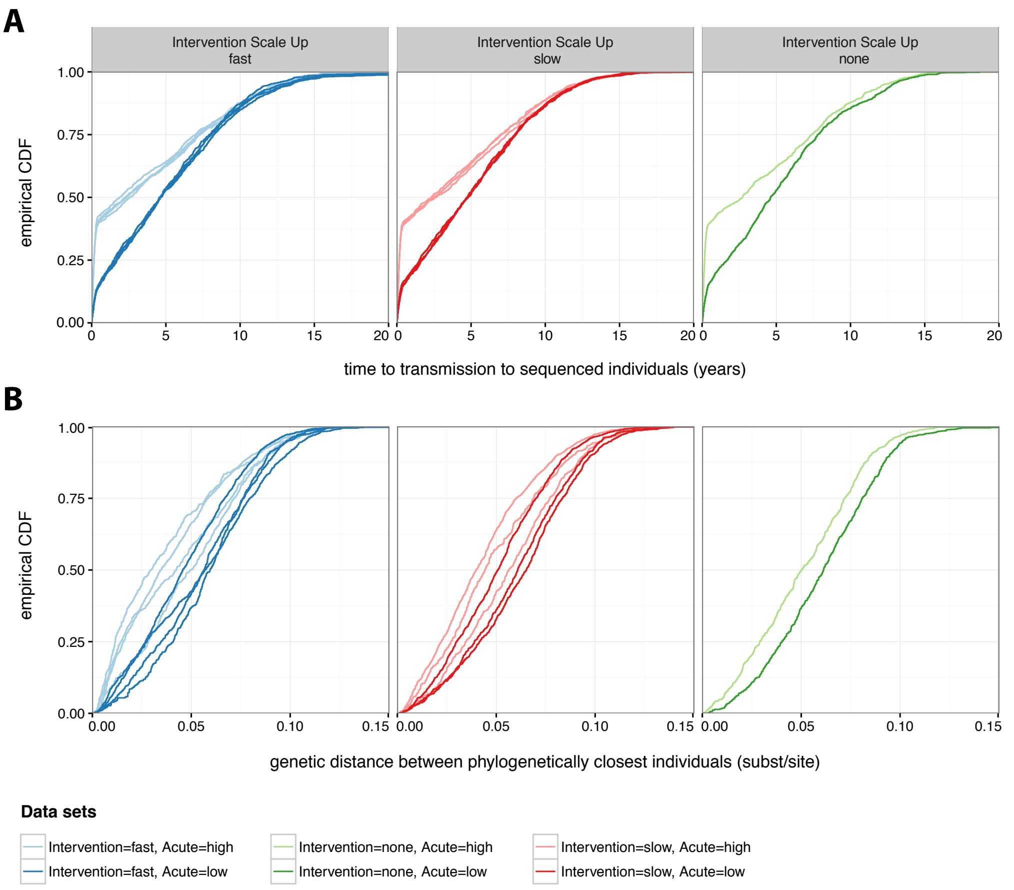

Figure S6. Signal to noise indicators for estimating the proportion of early transmission on the Regional simulations. Phylogenetic methods for estimating the proportion of early transmissions make use of information in branch length distributions, with shorter branches indicating faster transmission from individuals in early stages of infection. We report population-level indicators of differences in branch length distributions for the 10% and 40% Acute scenarios in the Regional simulations. Each line represents empirical cumulative distribution functions of a particular indicator, calculated on one simulation. Simulations are grouped into 10% and 40% Acute scenarios (darker and lighter lines) and intervention scenarios (color), to visualize signal versus noise. (**A**) Empirical CDF of the generation time distribution, among the subset of sequenced individuals. The generation time of sequenced individual was the time from infection of his transmitter to infection of the sequenced individual. High and low %Acute scenarios are clearly distinct from each other. The generation time was not known by participants. (**B**) In the simulations, generation times are reflected in the genetic distance between transmitters and recipients along the tree. However, not all transmitters appeared in the sequence data set. As a proxy, we considered the genetic distance between newly infected individuals and their genetically closest individual in the simulated data set. This proxy reflects information that was available to the participants. On average, high and low %Acute scenarios remained different from each other. This analysis suggests that some, but not strong, information exists in the Regional data sets for differentiating the 10% versus 40% Acute scenarios even at 8% sequence coverage.

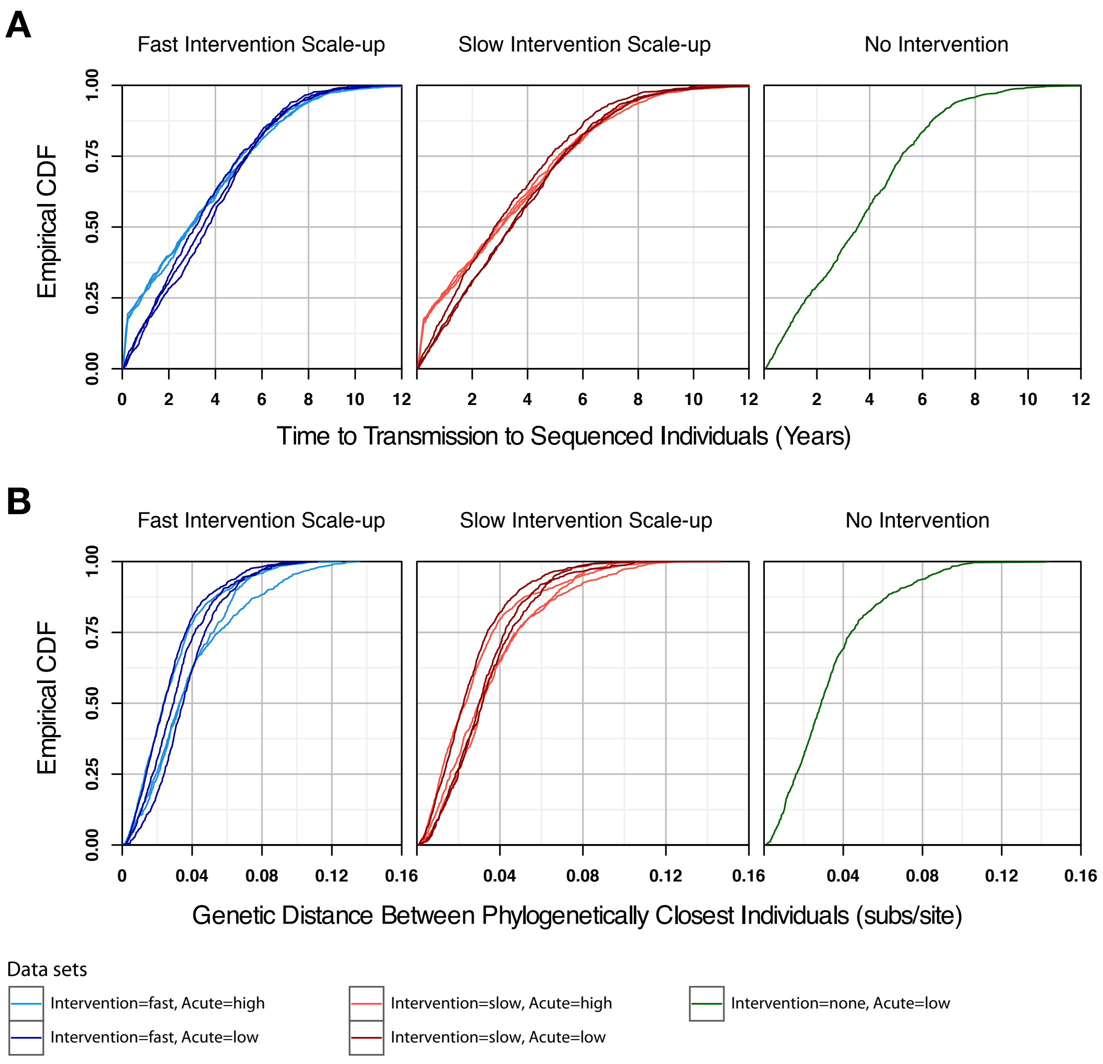

Figure S7. Signal to noise indicators for estimating the proportion of early transmission on the Village simulations. Phylogenetic methods for estimating the proportion of early transmissions make use of information in branch length distributions, with shorter branches indicating faster transmission from individuals in early stages of infection. We report population-level indicators of differences in branch length distributions for the 5% and 20% Acute scenarios in the Village simulations, see also figure S6. Each line represents empirical cumulative distribution functions of a particular indicator, calculated on one simulation. Simulations are grouped into 5% and 20% Acute scenarios (darker and lighter lines) and intervention scenarios (color), to visualize signal versus noise. (**A**) Empirical CDF of the generation time distribution, among the subset of sequenced individuals. The generation time of sequenced individual was the time from infection of his transmitter to infection of the sequenced individual. High and low %Acute scenarios are distinct from each other, although to lesser extent than for the Regional simulations. (**B**) In the simulations, generation times are reflected in the genetic distance between transmitters and recipients along the tree. We evaluated the extent to which the signal in (A) is still present at 25% and 50% sequence coverage, after transmission chains were collapsed to sampled individuals (transmitter may be lost) and then translated into viral trees. The plot shows, for all data sets, empirical CDFs of the genetic distances between phylogenetically closest individuals, as a proxy of transmission pairs. Unlike in (A), there is no apparent difference in high and low %Acute scenarios.

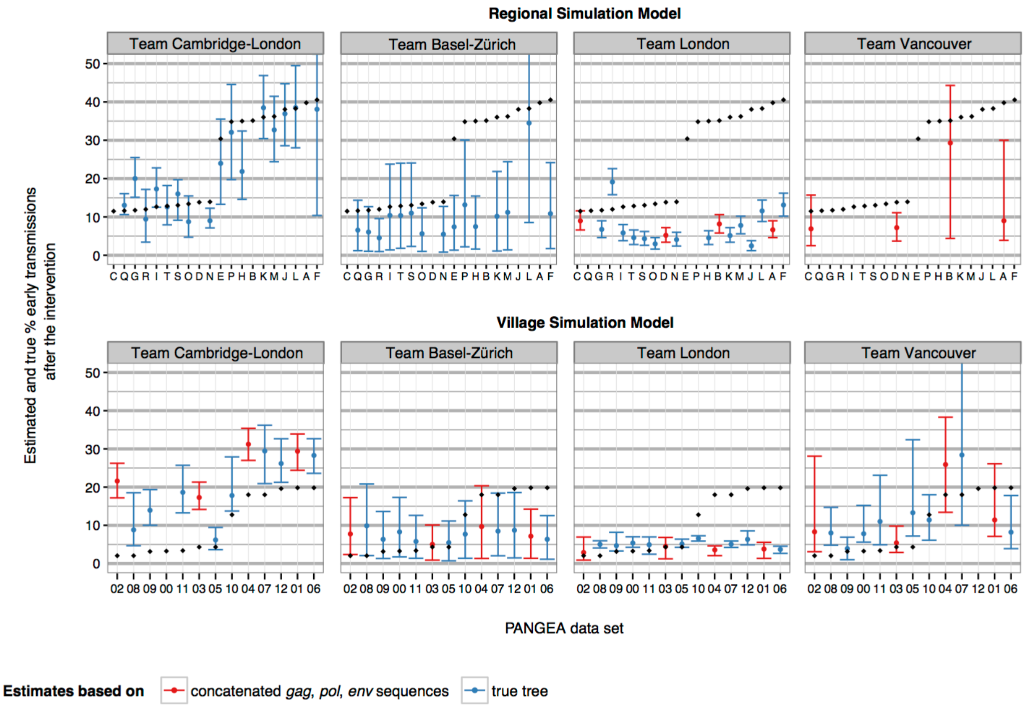

Figure S8 Estimates of the proportion of early transmissions after the intervention from phylogenetic methods on simulated PANGEA data sets. Submitted estimates are shown for each PANGEA data set by research team and model simulation (panels) and type of data provided (either sequences or the viral phylogenetic tree, color). Error bars correspond to 95% credibility or confidence intervals. True values are shown in black.

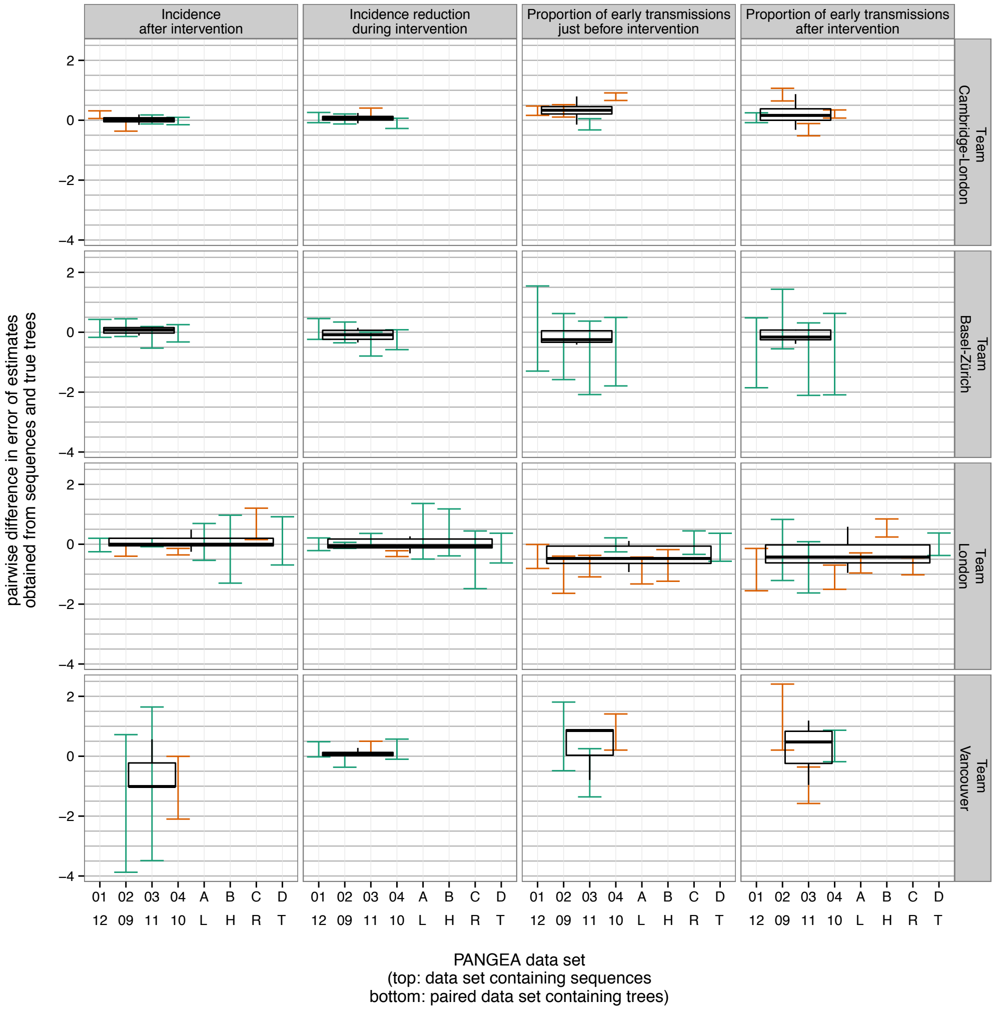

Figure S9. Differences in error of phylogenetic estimates obtained from sequence data, versus estimates obtained from sequence data and true phylogenetic trees known. PANGEA data sets containing sequences or true trees were considered, and paired if the underlying epidemiological scenario was identical (see x-axis and compare to table 3). For each objective, phylogenetic estimates $\hat{x}_i$ and true values x_i to each of these data sets were considered, and the error $e_i = log(\hat{x}_i) - log(x_i)$ was computed. The log scale was chosen so that errors were approximately normally distributed. The difference in errors $e_i - e_j$ between paired data sets i, j is plotted for each objective (columns) and each team (rows). Error bars indicate log transformed 95% confidence intervals; boxplots the distribution of central estimates; and significantly non-zero differences are highlighted in orange. Overall, phylogenetic estimates obtained from full genome sequence data sets were not significantly less accurate compared to estimates obtained with the true phylogenetic trees known (paired t-test: team Cambridge-London n=16, p=0.07; team Basel-Zürich n=16, p=0.79; team London n=32, p=0.033; team Vancouver n=13, p=0.87).

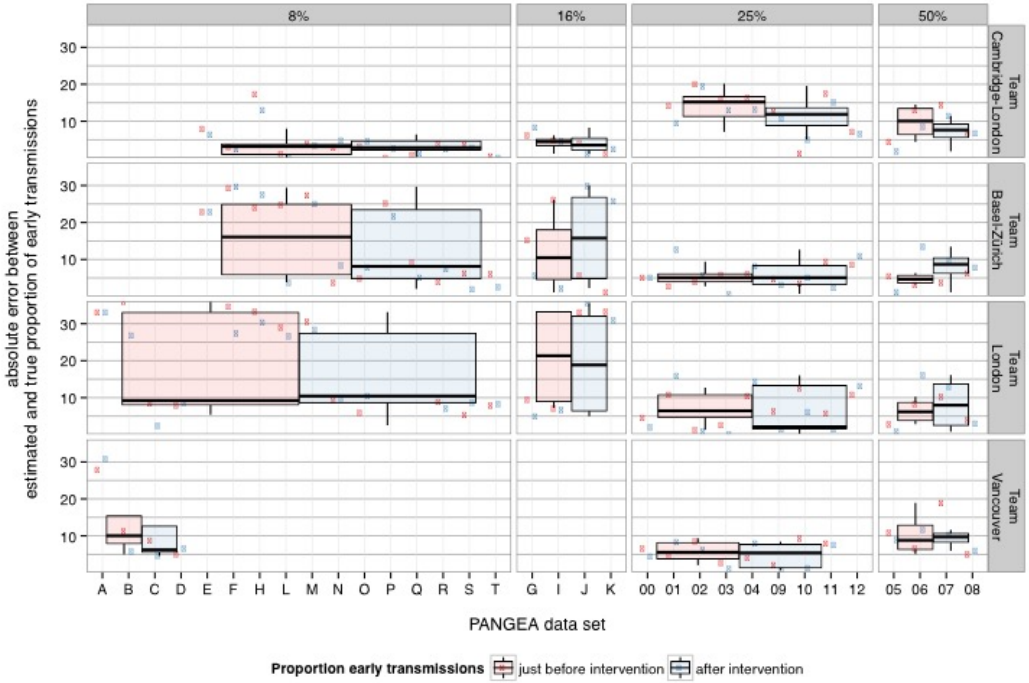

Figure S10. Accuracy of phylogenetic estimates of the proportion of early transmissions on simulated PANGEA data sets as a function of sampling coverage. For each PANGEA data set, the absolute error in the phylogenetic estimates of the proportion of early transmissions from individuals in their first three months of infection is shown by sequence coverage at the end of the simulation (panels). Each panel also compares the absolute error in estimates for the year just before the intervention (red) to that after the intervention (blue). Boxplots highlight the median absolute error and the interquartile range.

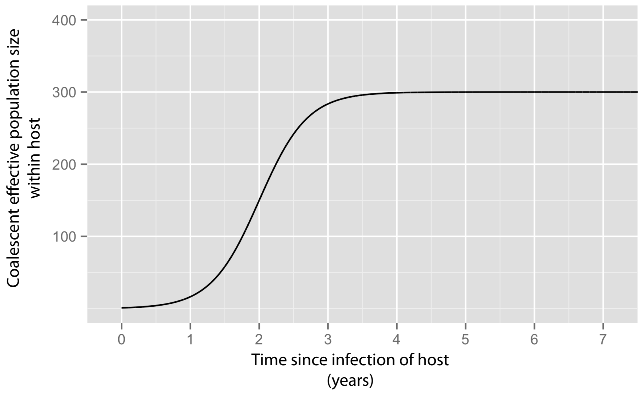

Figure S11. Within-host effective population size model of the Village and Regional simulations. Viral trees were generated under a hybrid within- and between-host coalescent model as described in tables S1 and S2, using the logistic effective population size model shown in this figure.

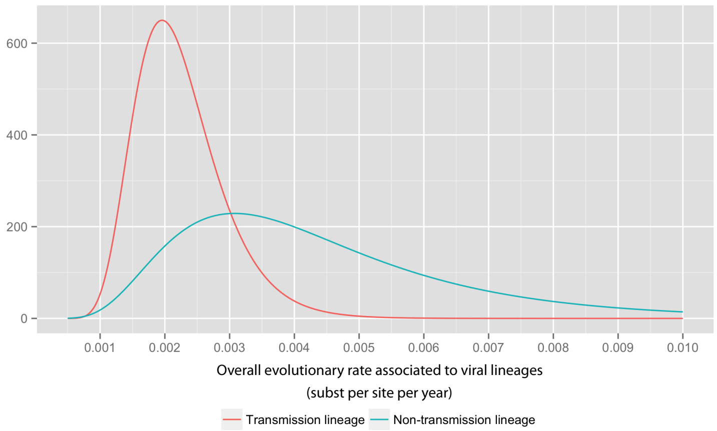

Figure S12. Sampling distribution of evolutionary rates of the Regional simulations. To simulate viral sequences along viral trees model as described in table S1, overall evolutionary rates were sampled from the log-normal distribution models shown in this figure and associated with transmission and non-transmission lineages of the viral tree. Sampled rates were used to translate branches into units of average substitutions per site per year.

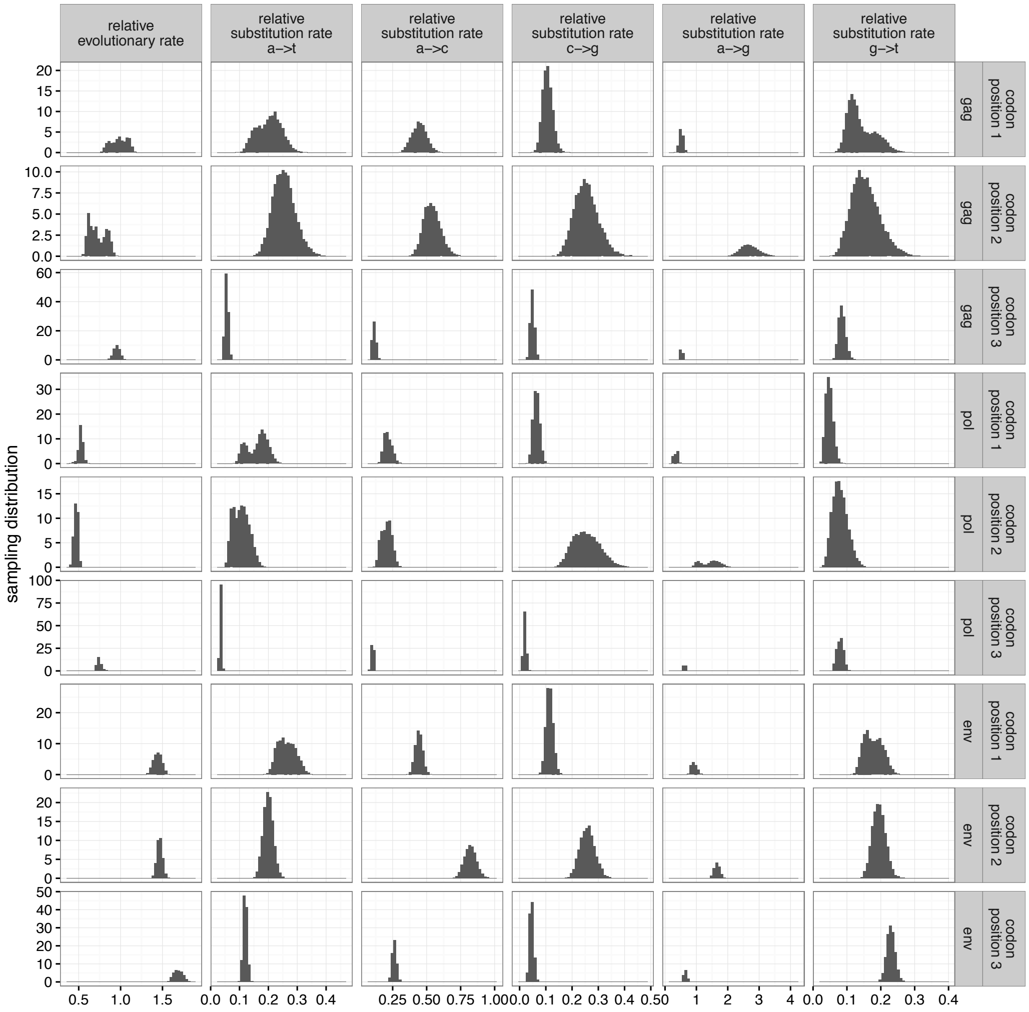

Figure S13. Sampling distribution of relative evolutionary rates and relative substitution rates of the Regional simulations. To simulate viral sequences along viral trees model as described in table S1, relative evolutionary rates by gene and codon position were sampled for each transmission chain as shown in this figure. The sampling distributions were obtained through BEAST phylogenetic analyses of full-genome HIV-1 subtype C sequences. GTR+Γ substitution models were used by gene and codon position, and relative substitution rates were sampled in the same manner. In the Village simulation, relative evolutionary rates and relative substitution rates were similar.

REFERENCES

1. Cori A, Ayles H, Beyers N, Schaap A, Floyd S, Sabapathy K, et al. HPTN 071 (PopART): a cluster-randomized trial of the population impact of an HIV combination prevention intervention including universal testing and treatment: mathematical model. PLoS One. 2014;9(1):e84511.
2. Hue S, Hassan AS, Nabwera H, Sanders EJ, Pillay D, Berkley JA, et al. HIV type 1 in a rural coastal town in Kenya shows multiple introductions with many subtypes and much recombination. AIDS research and human retroviruses. 2012;28(2):220-4.
3. Grabowski MK, Lessler J, Redd AD, Kagaayi J, Laeyendecker O, Ndyanabo A, et al. The role of viral introductions in sustaining community-based HIV epidemics in rural Uganda: evidence from spatial clustering, phylogenetics, and egocentric transmission models. PLoS Med. 2014;11(3):e1001610.
4. Cori A, Pickles M, van Sighem A, Gras L, Bezemer D, Reiss P, et al. CD4+ cell dynamics in untreated HIV-1 infection: overall rates, and effects of age, viral load, sex and calendar time. Aids. 2015;29(18):2435-46.
5. Hollingsworth TD, Anderson RM, Fraser C. HIV-1 transmission, by stage of infection. The Journal of infectious diseases. 2008;198(5):687-93.
6. Boily MC, Baggaley RF, Wang L, Masse B, White RG, Hayes RJ, et al. Heterosexual risk of HIV-1 infection per sexual act: systematic review and meta-analysis of observational studies. The Lancet infectious diseases. 2009;9(2):118-29.
7. Auvert B, Taljaard D, Lagarde E, Sobngwi-Tambekou J, Sitta R, Puren A. Randomized, controlled intervention trial of male circumcision for reduction of HIV infection risk: the ANRS 1265 Trial. PLoS Med. 2005;2(11):e298.
8. Bailey RC, Moses S, Parker CB, Agot K, Maclean I, Krieger JN, et al. Male circumcision for HIV prevention in young men in Kisumu, Kenya: a randomised controlled trial. Lancet. 2007;369(9562):643-56.
9. Gray RH, Wawer MJ, Polis CB, Kigozi G, Serwadda D. Male circumcision and prevention of HIV and sexually transmitted infections. Curr Infect Dis Rep. 2008;10(2):121-7.
10. Iwuji CC, Orne-Gliemann J, Tanser F, Boyer S, Lessells RJ, Lert F, et al. Evaluation of the impact of immediate versus WHO recommendations-guided antiretroviral therapy initiation on HIV incidence: the ANRS 12249 TasP (Treatment as Prevention) trial in Hlabisa sub-district, KwaZulu-Natal, South Africa: study protocol for a cluster randomised controlled trial. Trials. 2013;14:230.
11. Moore JS, Essex M, Lebelonyane R, El Halabi S, Makhema J, Lockman S, et al. Botswana Combination Prevention Project (BCPP) 2013 [Available from: https://clinicaltrials.gov/ct2/show/NCT01965470.
12. Hayes R, Ayles H, Beyers N, Sabapathy K, Floyd S, Shanaube K, et al. HPTN 071 (PopART): rationale and design of a cluster-randomised trial of the population impact of an HIV combination prevention intervention including universal testing and treatment - a study protocol for a cluster randomised trial. Trials. 2014;15:57.
13. Pybus OG, Rambaut A. Evolutionary analysis of the dynamics of viral infectious disease. Nat Rev Genet. 2009;10(8):540-50.
14. Didelot X, Gardy J, Colijn C. Bayesian inference of infectious disease transmission from whole-genome sequence data. Mol Biol Evol. 2014;31(7):1869-79.
15. Hall MD. Phylodynamics of infectious diseases of livestock: preparing for the era of large-scale sequencing: PhD thesis, University of Edinburgh; 2016.

16. Lemey P, Rambaut A, Pybus OG. HIV evolutionary dynamics within and among hosts. AIDS reviews. 2006;8(3):125-40.
17. Walker PR, Pybus OG, Rambaut A, Holmes EC. Comparative population dynamics of HIV-1 subtypes B and C: subtype-specific differences in patterns of epidemic growth. Infection, genetics and evolution : journal of molecular epidemiology and evolutionary genetics in infectious diseases. 2005;5(3):199-208.
18. Vrancken B, Rambaut A, Suchard MA, Drummond A, Baele G, Derdelinckx I, et al. The genealogical population dynamics of HIV-1 in a large transmission chain: bridging within and among host evolutionary rates. PLoS Comput Biol. 2014;10(4):e1003505.
19. Alizon S, Fraser C. Within-host and between-host evolutionary rates across the HIV-1 genome. Retrovirology. 2013;10.
20. Opio A, Muyonga M, Mulumba N. HIV infection in fishing communities of Lake Victoria Basin of Uganda--a cross-sectional sero-behavioral survey. PLoS One. 2013;8(8):e70770.
21. Seeley J, Nakiyingi-Miiro J, Kamali A, Mpendo J, Asiki G, Abaasa A, et al. High HIV incidence and socio-behavioral risk patterns in fishing communities on the shores of Lake Victoria, Uganda. Sexually transmitted diseases. 2012;39(6):433-9.
22. Fraser C, Hollingsworth TD, Chapman R, de Wolf F, Hanage WP. Variation in HIV-1 set-point viral load: epidemiological analysis and an evolutionary hypothesis. Proc Natl Acad Sci U S A. 2007;104(44):17441-6.
23. Heled J, Drummond AJ. Bayesian inference of species trees from multilocus data. Mol Biol Evol. 2010;27(3):570-80.
