# Supplementary material for: Phylogenetic Tools for Generalized HIV-1 Epidemics: Findings from the PANGEA-HIV Methods Comparison
Source: Mol Biol Evol. 2016 Oct 7;34(1):185–203. doi: 10.1093/molbev/msw217 (PMC5854118; doi:10.1093/molbev/msw217)
Supplement: Supplementary Data [file msw217_suppl.zip › MBE-16-0805_OliverRatmann_v160928_Text_S2.pdf]

Phylogenetic Tools For Generalized HIV-1 Epidemics: Findings from the PANGEA-HIV Methods Comparison

Supplementary Text S2: Information provided to participants – round 1

PANGEA-HIV (Phylogenetics and Networks for Generalised HIV Epidemics in Africa) is a major new initiative funded by the Bill and Melinda Gates Foundation to generate a large volume of next generation sequence data from African HIV cohorts to facilitate the phylodynamic characterization of generalized HIV epidemics.

The *PANGEA-HIV Methods Milestone 1* aims to evaluate existing phylogenetic methods in their ability to identify recent changes in HIV incidence in order to inform HIV prevention efforts in sub-Saharan Africa. Research groups are invited to participate in a blinded methods comparison exercise on simulated sequence data sets that capture different HIV transmission dynamics in generalized HIV-1 epidemics. Secondary aims of the exercise are to evaluate the merits of full genome sequence data, and the impact of changing sequence coverage.

With this exercise, *PANGEA-HIV* aims to direct further methods development in collaboration with participating research groups. Collaborative research teams will be formed to analyse the approximately 20,000 full genome HIV sequences with matched demographic and clinical patient data that are to be generated by PANGEA-HIV.

The PANGEA methods comparison working group, and *the PANGEA Consortium Executive group*

Table of Contents

PANGEA-HIV **2**

PANGEA Methodology Milestone 1 .... **3**

Introduction 3

Objectives 4

Collaborative blinded methods comparison 5

Research timelines and outputs 6

PANGEA – HIV

PANGEA-HIV (Phylogenetics and Networks for Generalised HIV Epidemics in Africa) is a major new initiative funded by the Bill and Melinda Gates foundation to

1. deliver ~20,000 full length HIV-1 gene sequences along with associated clinical and demographic patient covariates from several African cohort and study sites: the Botswana Combination Prevention Project (Botswana), the Africa Centre for Health and Population Studies at the University of KwaZulu-Natal (South Africa), the MRC/UVRI Uganda research unit on AIDS (Uganda), the Rakai Health Sciences Programme (Uganda), and HPTN071/ Popart (Zambia and South Africa).
2. direct the further development of phylogenetic and phylodynamic methods to address key challenges in measuring, understanding and controlling HIV transmission dynamics of generalised HIV epidemics

Central questions to be addressed with existing or new phylogenetic/phylodynamic methods in the context of the generalised HIV epidemics in sub-Saharan Africa are

1. *What can be inferred about epidemic dynamics and sexual network characteristics from phylogenetic and self-reported epidemiologic data? What does that imply for control strategies in local or regional settings where HIV prevalence is well in excess of 20% of the adult population?*
2. *What are the transmission dynamics of a generalized epidemic and how do they differ from those of a concentrated epidemic where data are already available?*
3. *What are, at the individual level, the characteristics of infectiousness? Can we identify individuals at greater risk of transmitting the virus, and should these be prioritized for frequent testing and immediate ART?*
4. *How does Next Generation Sequence HIV full genome data improve the inference of transmission dynamics?*

PANGEA Methodology Milestone 1

Introduction

Different phylogenetic and phylodynamic methods have been adopted to characterize concentrated HIV epidemics in Europe and the US, largely from partial HIV-1 pol sequences collected through local, regional or national HIV treatment monitoring studies. There is little consensus on the ability of the various methods to accurately

analyse declining, stable or increasing HIV epidemics at different scales, both in terms of geographical range and epidemic scale. Little is known on the power of the various methods in reliably assessing these HIV epidemics from data that differs in completeness and may be biased. This is particularly so for the application of these methods to HIV next generation sequencing data from generalised epidemics. We expect these methods to have - ultimately - profound implications to our understanding of HIV-1 transmission and our ability to prevent transmission. It is of critical importance to understand - now - the applicability and potential shortcomings of these methods to the kind of data that will be generated by the PANGEA consortium.

Objectives

Research groups are invited to participate in a blinded methods comparison exercise on simulated sequence data sets that capture different HIV transmission dynamics in generalized HIV-1 epidemics.

The primary objective of the *PANGEA-HIV Methods Milestone 1* is to evaluate existing phylogenetic methods in their ability to accurately and reliably identify changes in HIV incidence that might occur over a few years representing a community-based intervention in sub-Saharan Africa in the simulation.

Secondary objectives of the exercise are to evaluate

- improvements in accuracy and power through the use of concatenated HIV-1 *gag*, *pol* and *env* sequence data as compared to HIV-1 *pol* sequence data,
- accuracy and power with respect to different sequence sampling intensities.

Simulation scenarios

Generalised HIV-1 epidemics were simulated for a relatively small “Ugandan” village population of ~8,000 individuals and a larger “South African” regional population of ~40,000 individuals from two structurally different, agent-based epidemiological models. Different incidence scenarios and contamination scenarios (source cases from outside the study population) were simulated. Different proportions of the population were sampled. Each of these scenarios is tagged with a unique identifier (sc[A-Z]). Further details on the simulated data are available below.

Each data set consists of several hundred simulated HIV-1 subtype C viral sequences, comprising *gag*, *pol* and *env* sequences. Several highly variable genome regions were excluded in the simulation. The label of each sequence contains additional information on the individual ID, date of sequence sampling, date of birth if available, and gender.

For the “village” simulation, each scenario contains sequences from a short time period of 3 years. Data sets with the same epi and sample identifiers are sampled from the same epidemic at the same sampling fraction. For the “regional” simulation, each scenario contains sequences sampled for a longer time period spanning 40 years. Scenarios differ in HIV-1 incidence dynamics from a time point after the year 2000. 20 replicate data sets using different random number seeds (rep[1-20]) were generated to evaluate power.

Evaluation criteria

We ask participating research groups to address, where possible, the following questions.

For each scenario of the village simulation,

- Was the epidemic growing, stationary or declining?
- If the epidemic was not stationary, what was the growth / negative growth rate?
- What is the proportion of annual new HIV infections relative to the population at risk of HIV infection?

For each scenario and each replicate of the regional simulation,

- Was the epidemic growing, stationary or declining by the end of the simulation?
- In which calendar year did incidence start to change?
- If the epidemic was not stationary, what was the growth / negative growth rate by the end of the simulation?
- What is the proportion of annual new HIV infections relative to the population at risk of HIV infection by the end of the simulation?

Comparing scenarios of the village simulation with the same epi and sample identifier,

- How large is the relative change in incidence between scenarios?

Comparing scenarios of the regional simulation,

- How large is the relative change in incidence by the end of the simulation between the stationary scenario(s) and the changing incidence scenario(s)?

Please use the PANGEAHIVsim_EvaluationSheet to return your responses (on Dropbox). Where possible, please conduct two analyses, the first using the concatenated *gag+pol+env* genome and the second using only the *pol* gene.

Research timeline and outputs

- **7th November 2014**
  Deadline for early research reports. Using the PANGEAHIVsim_Report document on Dropbox as a template, please describe briefly the methods you are using or have developed and provide a short summary of your preliminary findings on up to 2-3 pages. Feedback to / from participating research groups as needed..
- **2nd December 2014**
  Workshop to compare and consolidate initial results in London, UK. Participating analysis groups to give summaries of progress and feedback regarding additional simulations.
- **End December 2014**
  Deadline for submission of analyses. The PANGEA steering committee will consolidate and communicate the findings jointly with the participating groups to report to the Bill and Melinda Gates foundation, and in a publication.
- **16th May 2015**
  Satellite meeting to bring together final results of simulation based collaboration at HIV Dynamics and Evolution meeting in Budapest, Hungary.

PANGEA-HIV methods comparison working group

In alphabetical order
Anne Cori [¶], Christophe Fraser [¶], Matthew Hall [*], Emma Hodcroft [*], Andrew Leigh Brown [*], Mike Pickles [¶], Andrew Rambaut [*], Manon Ragonnet-Cronin [*], Oliver Ratmann [¶]
[*]University of Edinburgh, United Kingdom
[¶]Imperial College London, United Kingdom

The data were generated by

- "African village" simulation: Emma Hodcroft
- "South African"-like regional simulation: Anne Cori, Mike Pickles
- HIV-1 *gag*, *pol* and *env* genome sequences: Matthew Hall, Oliver Ratmann

PANGEA Methodology Milestone 1 – Further details

South African regional simulation

The "South African" regional simulation scenarios were generated under an agent-based epidemiological model that has been developed as part of the HPTN 071 / PopART community randomized trial in South Africa and Zambia.

The epidemiological simulation starts in 1975 and ends in 2020. Individuals are stratified by gender, age, and level of sexual risk. Partnerships form and dissolve, with partner acquisition and concurrency depending on the sexual risk category. HIV transmissibility varies over the natural history of HIV by CD4 stages, acute/chronic HIV infection, circumcision status and condom use. Individuals within the simulated region have sexual partnerships with individuals outside the simulated region.

The viral molecular genetic simulation turns generated transmission chains into multiple phylogenies under a coalescent model that has within-host and between-host evolutionary components. The tips of the phylogeny correspond to sampling events. For each phylogeny, root sequences were generated from real sequences in the Los Alamos sequence database. Tip sequences were generated along the simulated phylogeny under a GTR site substitution model for the following genomic regions
1. gag: p17 start to pol PROT start; length 1440 nucleotides. The simulated gag gene does not include the last 14 amino acids of p6, due to the overlap with pol.
2. pol: PROT start to Integrase end; length 2844 nucleotides.
3. env: CDS signal peptide start to gp41 end; length 2523 nucleotides.

Three epidemiological scenarios A, B, C are generated, which differ in HIV-1 incidence dynamics from a time point after the year 2000. The following patient metavariables are available: Gender, Date of Birth (DOB), Date of Death (DOD), Time of sequence sampling (TIME_SEQ), CD4 count at time of sequence sampling (CD4_SEQ), Infected within one year of sequence sampling (INCIDENT_WITHIN1YEAR_SEQ).

Approximately 1,000 viral sequences are randomly sampled from HIV infected individuals between 1980 and 2020. Over time and across scenarios, the fraction of sampled sequences changes. Evolutionary simulation parameters are held fixed across scenarios.

FAQ

1. **How does the population size change over time?**
   The population size follows South African census estimates. In 1980, the population is a bit smaller than the census estimate, closer to ~ 20 million.
2. **Are there multiple introductions at the start of the simulation in 1975?**
   There are multiple introductions from outside the 'region', including the baseline year 1975. We generated the starting sequences based on phylogenetic estimates of the HIV introduction into South Africa, and expect TMRCA's before 1975.
3. **Are the viral lineages recombinating?**
   No, they are not – phew. ☺
4. **Is there ART in the model?**

Changing ART coverage is implicitly accounted for through changing transmission intensities.

5. **What has changed between the October and November simulations?**
We changed the way the sequences were sampled as the specification changed to include CD4 counts. So, different individuals with different population identifiers are now sampled. The epidemic model parameters remained exactly the same.

African Village simulation

For this scenario, an HIV epidemic was simulated in a population of ~8,000 individuals using an individual-based model from first introduction until incidence stabilised. The simulations were run for 70 years. Partnerships and contact rate depend on the gender and risk group of the individuals, with HIV transmissibility varying through acute, chronic, and AIDS stages. Individuals may have contact with individuals from villages outside the focal population.

Three similar HIV epidemic scenarios were simulated. From each simulation, samples were taken during 3 different time periods each lasting 3 years. Each of these time intervals corresponds to a period of increasing, decreasing or stationary incidence dynamics. Two different sampling fractions are represented among the simulations, with one of the 3 scenarios sampled at both fractions leading in total to 9 scenarios A, B, …, I.

The simulation is set to keep the population approximately constant. During the peak years of the epidemic the population declines by about 1% per year.

Participants will notice that the sample times for all scenarios have been blinded. First, meaningless years were used to avoid preconceived bias about what was happening in the HIV epidemic in Africa at any given real date. Second, the sample dates for each time point have been adjusted to avoid bias based on the relative timing of the samples in each. As in real life, participants do not know beforehand the current dynamics of the epidemic.

Because of this, combining the data from any of the separate samples will give erroneous results.

Each sequence is a concatenated sequence of gag, pol, and env. Gag runs from 1-1479bp, pol from 1480-4479, and env from 4480-6987. Each sequence is labelled with the user ID, gender, and sample date (in decimal-year format). User IDs are randomly assigned and meaningless.
