# Supplementary material for: Phylogenetic Tools for Generalized HIV-1 Epidemics: Findings from the PANGEA-HIV Methods Comparison
Source: Mol Biol Evol. 2016 Oct 7;34(1):185–203. doi: 10.1093/molbev/msw217 (PMC5854118; doi:10.1093/molbev/msw217)
Supplement: Supplementary Data [file msw217_suppl.zip › MBE-16-0805_OliverRatmann_v160928_Text_S3.pdf]

Phylogenetic Tools For Generalized HIV-1 Epidemics: Findings from the PANGEA-HIV Methods Comparison

Supplementary Text S3: Information for participants – round 2

PANGEA-HIV (Phylogenetics and Networks for Generalised HIV Epidemics in Africa) is a major new initiative funded by the Bill and Melinda Gates Foundation to generate a large amount of next generation sequence data and to provide phylogenetic tools to measure the impact of HIV prevention efforts in generalized epidemics in sub-Saharan Africa.

Research groups are invited to participate in a blinded methods comparison exercise on simulated HIV sequence data sets to test the performance of current phylogenetic methods before their application on real data.

The *PANGEA-HIV Methods Milestone 1* aims to evaluate current phylogenetic methods in their ability to identify recent changes in HIV incidence and the proportion of transmissions that originate from individuals in early HIV infection. Secondary aims of the exercise are to evaluate the merits of full genome sequence data, the impact of sequence coverage, and the impact of the proportion of transmissions originating from outside the study area. The simulation scenarios are challenging and capture detailed aspects of HIV transmission dynamics and intervention efforts that are typical for sub-Saharan Africa.

Based on the outcomes of this exercise, collaborative research teams will be formed to analyse the full genome HIV that are to be generated by PANGEA-HIV.

The PANGEA methods comparison working group, and *the PANGEA Consortium Executive group*

PANGEA – HIV

PANGEA-HIV (Phylogenetics and Networks for Generalised HIV Epidemics in Africa) is a major new initiative funded by the Bill and Melinda Gates foundation to

1. deliver a large volume of full length HIV-1 gene sequences along with associated clinical and demographic patient covariates from several African cohort and study sites: the Botswana Combination Prevention Project (Botswana), the Africa Centre for Health and Population Studies at the University of KwaZulu-Natal (South Africa), the MRC/UVRI Uganda research unit on AIDS (Uganda), the Rakai Health Sciences Programme (Uganda), and HPTN071/ Popart (Zambia and South Africa).
2. direct the further development of phylogenetic and phylodynamic methods to address key challenges in measuring, understanding and controlling HIV transmission dynamics of generalised HIV epidemics

PANGEA-HIV aims to address the questions

1. *What can be inferred about epidemic dynamics and sexual network characteristics from phylogenetic and self-reported epidemiologic data? What does that imply for control strategies in local or regional settings where HIV prevalence is well in excess of 20% of the adult population?*
2. *What are the transmission dynamics of a generalized epidemic and how do they differ from those of a concentrated epidemic where data are already available?*
3. *What are, at the individual level, the characteristics of infectiousness? Can we identify individuals at greater risk of transmitting the virus, and should these be prioritized for frequent testing and immediate ART?*
4. *How does Next Generation Sequence HIV full genome data improve the inference of transmission dynamics?*

PANGEA HIV Methods Milestone 1

Introduction

Phylogenetic methods have been widely applied to characterize concentrated HIV epidemics such as Europe and the US, but not in the context of generalized HIV epidemics in sub-Saharan Africa. These analyses have been based on partial HIV sequence data, but not on full genome sequence data. It is not known how current methods are best scaled to full genome sequence data, and if they can accurately uncover aspects of HIV transmission dynamics from generalized HIV epidemics under typical sequence sampling conditions. We expect these methods to have - ultimately - profound implications to our understanding of HIV-1 transmission and our ability to prevent transmission. It is of critical importance to understand - now - the applicability and potential shortcomings of these methods to the kind of data that will be generated by the PANGEA consortium.

To assess the performance of current phylogenetic methods in a controlled setting, the PANGEA HIV methods comparison working group implemented two highly detailed epidemiological and evolutionary models of generalized HIV epidemics to simulate full genome HIV sequences and phylogenetic trees. The simulation scenarios are designed to capture central aspects of partnering HIV prevention study sites, e.g. the Treatment as Prevention (TasP) trial in South Africa.

Objectives

Research groups are invited to participate in a blinded methods comparison exercise on simulated sequence data sets and simulated phylogenetic trees to address the following specific objectives.

Primary objectives
To evaluate existing phylogenetic methods in their ability to measure

1. changes in HIV incidence that might occur over a few years representing a community-based intervention in sub-Saharan Africa in the simulation. The outcome measure is annual HIV incidence in % of the number of individuals that are at risk of HIV infection.

2. the proportion of HIV transmissions arising from individuals in early HIV infection at the start of the community intervention. The outcome measure is the proportion of new HIV cases from those in early HIV infection in the year before the start of the community intervention.

Secondary objectives
To evaluate

3. improvements through the use of concatenated HIV-1 *gag*, *pol* and *env* sequence data as compared to HIV-1 *pol* sequence data. The outcomes measure is the accuracy in answering the primary objectives.
4. the impact of sequence sampling coverage. The outcomes measure is the accuracy in answering the primary objectives.
5. the impact of the proportion of transmissions that occur from outside the study area. The outcomes measure is the accuracy in answering the primary objectives.

Overview of simulation models

Generalised HIV-1 epidemics were simulated for a relatively small "Ugandan" village population of ~8,000 individuals and a larger "South African" regional population of ~80,000 individuals from two structurally different, agent-based epidemiological models.

The regional simulation captures individual-level HIV transmission dynamics in a larger regional population that is broadly similar to a site (cluster) of the HPTN071/PopART HIV prevention trial in South Africa. Standard of care improved according to national guidelines over time. In a subset of simulations, an additional comprehensive HIV prevention combination package started in 2015 for three years, broadly similar to the HPTN071/PopART intervention. Since 2015, the population is monitored more actively, resulting in a moderate sampling coverage at a large scale (<10% of the infected population). Contamination through transmission from outside the regional area occurs in the range of available estimates for sub-Saharan Africa. More information is available in the appendix 'Regional simulation'.

The village simulation captures individual-level HIV transmission dynamics in a small village population. An intervention campaign started at some point after the epidemic peaked, and was followed for a long period of time. Some time after intervention was started, the simulated campaign was intensely monitored for three years, resulting in a relatively high sampling coverage at a small scale (>10% of the infected population). There is no additional intervention. Contamination through transmission from outside the village area is minimal. More information is available in the appendix 'Village simulation'.

Overview of simulation scenarios

Data sets were simulated from both models. Phylogenetic inference is often computationally expensive. To ease the computational requirements, the simulated phylogeny is provided for a subset of simulations that is of secondary importance. To address the primary objectives, parameters relating to HIV transmission dynamics and the efficacy of the prevention campaigns were varied for each model. Sequence data was generated. To address the

secondary objectives, parameters relating to the sampling frame and the proportion of transmissions from outside the study area were varied. Phylogenies were generated. Additional data for each sequenced individual, and additional population surveys on the course of the HIV epidemic until 2015 are available for each scenario. Please see the appendices for further information.

Primary Evaluation criteria

We aim to evaluate HIV transmission dynamics around an evaluation period that is close to the end of the simulation. For the village simulation, the evaluation period coincides with the intensely sampled period (see appendix). For the regional simulation, the evaluation period starts in January 2015 and ends one year before the end of the simulation, in almost all cases December 2019.

For each data set containing simulated sequences:

i. During the evaluation period, was incidence stable, declining or increasing? Please provide answers in terms of

$$'stable', 'declining', 'increasing'$$

ii. What is the annual % incidence in the last year of the evaluation period? Please provide answers in % incidence,

$$\%INC_{t\_e} = \frac{INC_{t\_e}}{S_{t\_e}},$$

where t_e is the last year of the evaluation period, INC_{t_e} is the estimated number of new cases in year t_e, and S_{t_e} is the estimated number of sexually active individuals that have not been infected until t_e.

iii. Comparing the year preceding the evaluation period to the last year of the evaluation period, what is the ratio in annual % incidence? Please provide answers in terms of

$$Ratio = \frac{\%INC_{t\_e}}{\%INC_{t\_s}}$$

where t_s denotes the year preceding the study period.

iv. Was the proportion of transmissions that originated from individuals in early HIV infection in the year preceding the evaluation period below 10%, between 10-30%, or above 30%? Please provide answers in terms of

$$' < 10\%', '10 - 30\%', ' > 30\%'$$

Here, early HIV infection is understood as the first 3 months after HIV infection.

v. What is the proportion of transmissions that originated from individuals in early HIV infection in the year preceding the evaluation period? Please provide answers in terms of

$$\%Early_{t\_s} = \frac{INC_{t\_s}(from\ early)}{INC_{t\_s}}$$

vi. What is the proportion of transmissions that originated from individuals in early HIV infection in the last year of the evaluation period? Please provide answers in terms of

$$\%Early_{t\_e} = \frac{INC_{t\_e}(from\ early)}{INC_{t\_e}}$$

Secondary Evaluation criteria

For each of the above data set, please report the outcome measures in (i) to (vi) above for two analyses:

vii. Using only the *pol* sequences

viii. Using the concatenated gag+pol+env sequences.

For each of the data sets containing a simulated phylogeny, please also report the outcome measures in (i) to (vi).

These simulation scenarios may vary from those used to evaluate the primary objectives in terms of

- The sequence sampling coverage
- When sequences are sampled during the course of infection
- The annual proportion of transmissions that originate from outside the study area.

Other Evaluation criteria

We encourage participants to fine-tune their phylogenetic methods to address the above outcome measures. These will be given preference in the methods comparison, presentations and publications. The methods comparison group may consider supplementary outcome measures provided by participants, if these can be directly calculated on the simulated data. Please note that effective population sizes or reproduction numbers cannot be calculated from the agent-based simulations.

Reporting

We will make evaluation sheets available as for the training round of the methods comparison exercise.

Thank you

The PANGEA-HIV methods comparison working group would like to thank all participants for their interest and contributions thus far.

Timelines

February 2015	Release of simulated sequence data sets and simulated phylogenies
27.02.2015	Presentation of interim results based on the training round at CROI
06.05.2015	Deadline for submission of analyses
13.05.2015	HIV Dynamics and Evolution 2015, where we will present an overview of the results of the methods comparison exercise. Individual submissions from participants are encouraged.
16.05.2015	PANGEA-HIV satellite meeting to present and discuss final results of the exercise in detail. All participating teams will have the opportunity to present their work.

PANGEA-HIV methods comparison working group

Leads: Christophe Fraser, Oliver Ratmann (Imperial College London); Andrew Leigh Brown, Emma Hodcroft (Edinburgh) Contributors: *Mike Pickles*, *Anne Cori* (Imperial College London)*; Matthew Hall, Samantha Lycett, Manon Ragonnet-Cronin, Gonzalo Yebra, Andrew Rambaut (University of Edinburgh)*
